# Analysis of the Metabolic Characteristics of Serum Samples in Patients With Multiple Myeloma

**DOI:** 10.3389/fphar.2018.00884

**Published:** 2018-08-22

**Authors:** Haiwei Du, Linyue Wang, Bo Liu, Jinying Wang, Haoxiang Su, Ting Zhang, Zhongxia Huang

**Affiliations:** ^1^MOH Key Laboratory of Systems Biology of Pathogen, Institute of Pathogen Biology, Chinese Academy of Medical Sciences & Peking Union Medical College, Beijing, China; ^2^Department of Hematology, Multiple Myeloma Medical Center of Beijing, Beijing Chao-yang Hospital, Capital Medical University, Beijing, China

**Keywords:** multiple myeloma, diagnosis, QExactiveTM Orbitrap MS, metabolome, biomarkers

## Abstract

**Aims:** This study aimed to identify potential, non-invasive biomarkers for diagnosis and monitoring of the progress in multiple myeloma (MM) patients.

**Methods:** MM patients and age-matched healthy controls (HC) were recruited in Discovery phase and Validation phase, respectively. MM patients were segregated into active group (AG) and responding group (RG). Serum samples were collected were conducted to non-targeted metabolomics analyses. Metabolites which were significantly changed (SCMs) among groups were identified in Discovery phase and was validated in Validation phase. The signaling pathways of these SCMs were enriched. The ability of SCMs to discriminate among groups in Validation phase was analyzed through receiver operating characteristic curve. The correlations between SCMs and clinical features, between SCMs and survival period of MM patients were analyzed.

**Results:** Total of 23 SCMs were identified in AG compared with HC both in Discovery phase and Validation phase. Those SCMs were significantly enriched in arginine and proline metabolism and glycerophospholipid metabolism. 4 SCMs had the discriminatory ability between MM patients and healthy controls in Validation phase. Moreover, 12 SCMs had the ability to discriminate between the AG patients and RG patients in Validation phase. 10 out of 12 SCMs correlated with advanced features of MM. Moreover, 8 out of 12 SCMs had the negative impact on the survival of MM. 5′-Methylthioadenosine may be the only independent prognostic factor in survival period of MM.

**Conclusion:** 10 SCMs identified in our study, which correlated with advanced features of MM, could be potential, novel, non-invasive biomarkers for active disease in MM.

## Introduction

Multiple myeloma (MM) is a lethal plasma cell malignancy characterized by the amplification of clonal plasma cells in the bone marrow (Hanbali et al., 2017). It accounts for 10% of hematologic malignancies worldwide, with clinical features consisting of hypercalcemia (C), renal failure (R), anemia (A), and bone lesions (B), which is also known as CRAB symptom (symptomatic MM or active MM) (Hanbali et al., 2017; Zhang et al., 2017).

Despite advances in the understanding of MM pathogenesis and improvements in treatment strategies of MM over the past decade, there are still the following problems in clinical practice. The incidence of MM is increasing year by year, but most of the patients have been in the middle or late stages of the disease when diagnosed. In addition, MM is incurable, and most of patients inevitably undergo disease progression or multiple relapses during the course. It is reported that the prognostic factors of MM include international staging system (ISS) stage or revised R-ISS staging, genetic aberrations and monoclonal protein (M protein) levels (Caltagirone et al., 2014; Kraj, 2014; Kuiper et al., 2015; Song et al., 2015; Tandon et al., 2017).

Relative to invasive detection methods such as puncture or biopsy of bone marrow, the ideal biomarker in clinical diagnosis of newly-diagnosed MM (NDMM) patients and the monitoring of relapsed MM (RMM) should possess optimal sensitivity and specificity in samples obtained from patients in a minimally-invasive manner, such as blood or saliva. The mounting evidence demonstrate that metabolomic profiling methods might be feasible for identifying diagnostic or prognostic biomarkers for patients with solid tumors and hematologic malignancies, including colorectal cancer, bladder cancer, acute myeloid leukemia and acute lymphoblastic leukemia (Bannur et al., 2014; Chen et al., 2014; Liu et al., 2017; Tan et al., 2017). Metabolomics is the quantitative measurement of all low-molecular-weight metabolites in an organism under specific environmental conditions, representing the end products of cellular processes (Jordan et al., 2009).

In our work, an ultraperformance liquid chromatography coupled with high-resolution orbitrap mass spectrometry, Q Exactive™ (UPLC-MS) was used to profile the serum of MM patients with active MM, responding MM and healthy controls. Differences in metabolomics data from the three groups were characterized by principal component analysis (PCA). Our study aimed to identify potentially non-invasive metabolic biomarkers for diagnosis and monitoring of the progress of MM.

## Methods

### Patients and samples

A total of 36 subjects were recruited into Discovery phase, including 22 patients with MM and 14 age-matched healthy individuals. A total of 75 subjects were recruited into Validation phase, including 55 MM patients and 20 age-matched healthy individual. The serum samples of Discovery phase and Validation phase were collected for metabolome analysis. The basic characteristics and clinical features of MM patients were recorded, including age, gender, stage of R-ISS, plasma cell, bone lesions, calcium, creatinine, hemoglobin.

The MM patients diagnosed at Beijing Chao-yang Hospital (western campus), Capital Medical University between October 2016 and November 2016. The diagnosis and response criteria were based on the International Myeloma Working Group diagnostic criteria (Rajkumar, 2016).

Conforms to the following criteria for the diagnosis of symptomatic myeloma (or active MM) by the International Myeloma Working Group (IMWG): more than 10% of clonal plasma cells in bone marrow and resulting in one of the CRAB symptoms of elevated serum calcium (Calcium > 2.75 mmol/L), kidney damage (Creatinine > 173 mmol/L), anemia (Hemoglobin < 10 g//L), and osteolytic bone destruction or pathological fracture. MM is a disease of repeated relapse patterns that is currently incurable. A cycle of total therapy for treatment consisted of induction remission of 4–6 courses of chemotherapy for newly diagnosed MM (NDMM) patients (in order to reduce tumor burden), then maintenance therapy (further reduce minor residual tumor) and induction remission after relapse.

The efficacy assessment after treatment needs to meet the following 2014 IMWG efficacy criteria for MM: complete response (CR): negative serum and urine immunofixation electrophoresis, plasma cells in bone marrow <5%; very good partial response (VGPR): serum and urine immunofixation electrophoresis is positive or serum M protein is reduced by ≥90% and urine M protein is <100 mg/24 h; partial response (PR): serum M protein reduction is reduced by ≥50% and urine M protein is <200 mg/24 h. The therapeutic efficacy should be at least above the PR after induction chemotherapy, in which M protein is the main criteria for MM efficacy evaluation. Disease progression refers to an increase in the level of M protein by more than 25%. If a patient develops new or severe CRAB symptoms after CR or PR, which is called symptomatic relapse, immediate induction chemotherapy should be performed.

In the current study, these symptomatic MM or active MM patients received initial frontline therapy with a bortezomib-based or non bortezomib-based regimen for average 4–6 cycles. While achieving a CR or PR, the regimen was repeated for two to four cycles or patients received autologous stem cell transplantation as consolidation therapy. In the absence of any response, the treatment regimen was modified. Maintenance therapy when utilized was with thalidomide 100 mg/day (An et al., 2015).

These MM patients were divided into two groups: active group (AG) and responding group (RG). Enrollment criteria: (1) active group (AG): symptomatic myeloma patients who currently have active CRAB symptoms need received induction chemotherapy, and were classified into active group (AG), including newly diagnosed MM (NDMM) and relapsed MM (RMM) patients. All patients had serum samples collected before initiation of any kind of treatment, and stored at −80°C for metabolism research. (2) responding group (RG): After an average of 4 courses of initial frontline induction chemotherapy, the patients were evaluated for therapeutic efficacy. Those patients with improvement of CRAB symptoms and the decrease of the M protein level was more than 50%, achieved PR at least were divided into responding group (RG). Serum was obtained from patients on day 21 after the fourth cycle and was stored at −80°C for metabolomic analyses. Exclusion criteria: (1) MM patients with insufficient chemotherapy for 4 courses; (2) patients with a decrease of M protein level less than 50% or insufficient to achieve PR at least.

**Figure 1:**
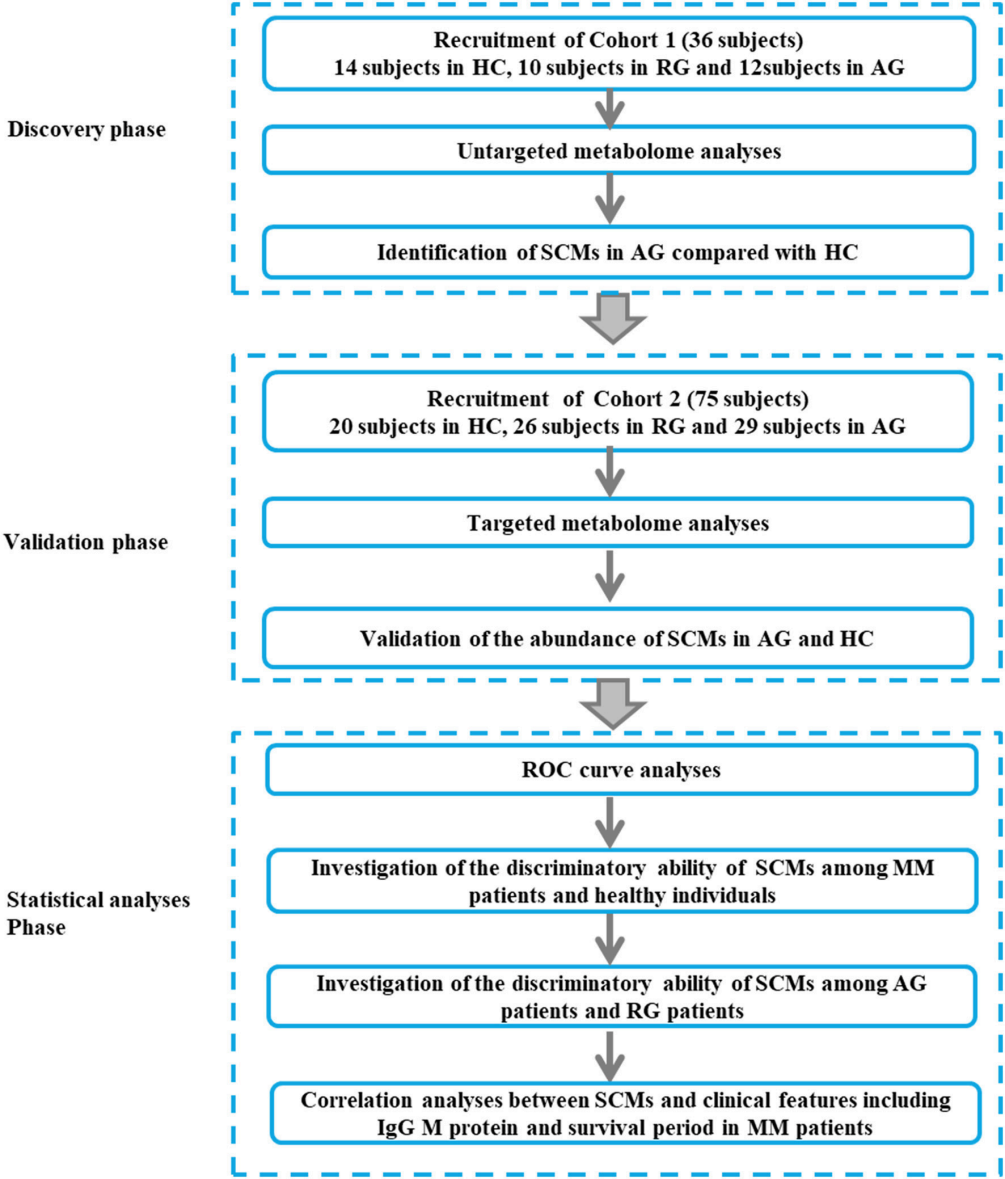
The workflow of our work. The Discovery phase (Cohort 1, 36 subjects), Validation phase (Cohort 2, 75 subjects) and Statistical (Cohort 2, 75 subjects) analyses phase were incorporated into our study. MM, multiple myeloma; AG, active MM patients also named as symptomatic myeloma; RG, MM patients responding to chemotherapy; HC, healthy controls.

The baseline clinical data of all 22 MM patients in Discovery phase and 55 patients in Validation phase were shown in Tables 1, 2. The workflow of our work was shown in Figure 1.

**Table 1:** Baseline clinical data of 22 cases of MM patients in Cohort 1.

**Characteristics**	**Overall**	**Groups**		***P*-value**
		**Active group (AG)**	**Responding group(RG)**	
Numbers	22	12(NDMM 4/RMM 8)	10	
Age (years)	61.1	63.5	57.9	0.223
Gender: M/F	14/8	8/4	6/4	0.746
**TYPE OF MM**				
Ig G(%))/Ig A/Ig D/κ/λ/NS	12(54.5%) /3/2/2/3/0	6(50.0%)/1/2/1/2/0	6(60.0%)/2/0/1/1/0	0.644
Stage of R-ISS: I/II/III	3(14%)/11(50%)/8(36%)	1(8%)/5(42%)/6(50%)	2(20%)/6(60%)/2(20%)	0.323
MM with EMP	3(13.6%)	2(16.7%)	1(10.0%)	0.650
**HIGH-RISK CYTOGENETICS**				
FISH del(17p)/1q21/IGH	3/4/9(*n* = 18)	2/4/4(*n* = 10)	1/0/5(*n* = 8)	0.159
Plasma cell (%)	12.5 ± 3.7	21.6 ± 45.6	31.5 ± 0.4	0.001
Calcium > 2.75 mmol/L	1	1	0	0.350
Creatinine > 177 mmol/L	3(13.6%)	3(25.0%)	0(0.0%)	0.089
Hemoglobin < 10 g//L	8(36.4%)	7(58.3%)	1(10.0%)	0.019
Bone lesions	17(77.3%)	10(83.3%)	7(70.0%)	0.457
LDH > 240 U/L	12(54.5%)	8(66.7%)	4(40.0%)	0.211
β_2−_MG > 5.5 mg/dl	6(27.3%)	6(50.0%)	0(0.0%)	0.009
Albumin < 35 g/L	5(22.7%)	3(25.0%)	2(20.0%)	0.781
Calcium (mmol/L)	3.2 ± 0.9	4.0 ± 0.5	2.2 ± 0.05	0.300
Creatinine (umol/L)	149.5 ± 38.0	214.8 ± 63.9	62.7 ± 12.7	0.004
Hemoglobin (g/L)	107.4 ± 6.3	93.2 ± 8.3	124.5 ± 46.4	0.043
Globulin (g/L)	31.7 ± 3.3	38.1 ± 5.3	23.7 ± 1.3	0.001
Amount of IgG (g/L)	27.8 ± 5.6	38.5 ± 7.0	15.9 ± 3.8	0.018
TC (mmol/L)	4.6 ± 0.2	4.6 ± 0.3	4.7 ± 0.3	0.922
TG (mmol/L)	2.3 ± 0.4	2.2 ± 0.5	2.5 ± 0.6	0.631
HDL-C (mmol/L)	1.0 ± 0.07	0.9 ± 0.05	1.1 ± 0.06	0.165
LDL-C (mmol/L)	2.6 ± 0.1	2.3 ± 0.2	2.9 ± 0.2	0.055

*M, male; F, female; MM, multiple myeloma; NDMM, newly-diagnosed patients with MM; RMM, relapsed patients with MM; R-ISS, Revised International Staging System; FISH, fluorescence in situ hybridization; LDH, Lactate dehydrogenase; β2-MG, β2-microglobulin; TC, total cholesterol; TG, triglyceride; HDL-C, high-density lipoprotein cholesterol; LDL-C, low-density lipoprotein cholesterol; Ig G, immunoglobulin G; Ig A, immunoglobulin A; Ig D, immunoglobulin D; NS, non-secretory*.

**Table 2:** Baseline clinical data of 55 cases of MM patients in Cohort 2.

**Characteristics**	**Overall**	**Groups**		***P*-value**
		**Active group (AG)**	**Responding group(RG)**	
Numbers	55	29(NDMM12/RMM17)	26	
Age (years)	61.5	61.7	61.3	0.861
Gender: M/F	33/22	17/12	16/10	0.825
**TYPE OF MM**				
Ig G(%))/Ig A/Ig D/κ/λ/NS	25(45.5%)/11/4/6/7/2)	14(48.3%)/5/3/3/3/1	11(42.3%)/6/1/3/4/1	0.921
Stage of R-ISS: I/II/III	7(13%)/18(33%)/30(55%)	2(7%)/7(24%)/20(69%)	5(19%)/11(42%)/10(39%)	0.092
MM with EMP	5(9.1%)	3(10.3%)	2(7.7%)	0.733
**HIGH-RISK CYTOGENETICS**				
FISH del(17p)/1q21/IGH	5/19/23(*n* = 40)	3/13/10(*n* = 22)	2/6/13(*n* = 18)	0.263
Plasma cell (%)	17.3 ± 2.8	31.1 ± 4.0	3.3 ± 0.9	0.000
Calcium > 2.75 mmol/L	2	2	0	0.173
Creatinine > 177 mmol/L	5(9.1%)	4(13.8%)	1(3.8%)	0.200
Hemoglobin < 10//L	21(38.2%)	17(58.6%)	4(15.4%)	0.001
Bone lesions	17(77.3%)	27(83.3%)	23(70.0%)	0.550
LDH > 240 U/L	6(10.9%)	6(20.7%)	0(0.0%)	0.014
β_2−_MG > 5.5 mg/dl	15(27.3%)	14(48.3%)	1(3.8%)	0.000
Albumin < 35 g/L	20(36.4%)	13(44.8%)	7(26,9%)	0.168
Calcium (mmol/L)	2.6 ± 0.3	3.0 ± 0.7	2.2 ± 0.03	0.264
Creatinine (umol/L)	124.3 ± 21.1	157.8 ± 35.1	86.8 ± 19.4	0.042
Hemoglobin (g/L)	104.1 ± 3.9	89.3 ± 5.1	120.6 ± 4.0	0.000
Globulin (g/L)	31.7 ± 2.4	38.2 ± 4.1	23.9 ± 1.0	0.004
Amount of IgG (g/L)	28.8 ± 3.5	38.4 ± 3.9	13.9 ± 1.7	0.000
TC (mmol/L)	4.3 ± 0.2	4.0 ± 0.3	4.7 ± 0.2	0.021
TG (mmol/L)	1.8 ± 0.2	1.7 ± 0.2	2.0 ± 0.3	0.446
HDL-C (mmol/L)	1.1 ± 0.04	1.0 ± 0.05	1.2 ± 0.06	0.009
LDL-C (mmol/L)	2.3 ± 0.1	2.1 ± 0.2	2.6 ± 0.1	0.005

*M, male; F, female; MM, multiple myeloma; NDMM, newly-diagnosed patients with MM; RMM, relapsed patients with MM; R-ISS, revised International Staging System; FISH, fluorescence in situ hybridization; LDH, lactate dehydrogenase; β_2_-MG, β_2_-microglobulin; TC, total cholesterol; TG, triglyceride; HDL-C, high-density lipoprotein cholesterol; LDL-C, low-density lipoprotein cholesterol; Ig G, immunoglobulin G; Ig A, immunoglobulin A; Ig D, immunoglobulin D; NS, non-secretory*.

### Ethics

This work was approved by the Ethics Committee of Beijing Chao-yang Hospital, Capital Medical University, and informed written consent was obtained from all patients and healthy individuals. The research complied with the principles of the Declaration of Helsinki.

### Serum sample preparation

For AG patients, serum samples were collected before 4 courses of first-line of anti-myeloma therapy and for RG patients, serum samples were collected after 4 courses of anti-myeloma therapy. The diagnosis criteria and treatment measurement of MM patients were shown as abovementioned. The collected serum samples were immediately frozen at −80°C for metabolomic analyses.

Metabolites were extracted from 100 μL of serum using 300 μL of acetonitrile, thoroughly mixed on a vortex mixer for 15 s, three times, and centrifuged at 12,000 rpm for 5 min at 4°C. For ultra high performance liquid chromatography analysis, 100 μL of supernatant was pipetted into vials to be analyzed on the UPLC-MS instrument.

### Non-targeted metabolomics analyses based on UPLC-MS

Chromatographic separation was performed on a Thermo Scientific™ Dionex™ UltiMate™ 3000 Rapid Separation LC (RSLC) system (Thermo Fisher Scientific, Waltham, MA, USA), equipped with a reversed-phase C_18_ column (HSS T3 column, 1.7 μm, 100 × 2.1 mm, Waters Corporation, Milford, MA, USA) or hydrophilic interaction liquid chromatography (HILIC) column (BEH Amide column, 1.7 μm, 100 × 2.1 mm, Waters Corporation, Mifold, MA, USA).

For lipid separation on a C_18_ column, mobile phase A consisted of 0.1% formic acid in water and mobile phase B consisted of 0.1% formic acid in methanol. The column temperature was set to 45°C. The flow rate was 300 μL/min and the injection volume was 1 μL. The gradient conditions for C_18_ separation of lipid are shown in Table S1.

For polarity separation of components on a HILIC column, mobile phase A consisted of 0.1% formic acid and 10 mmol/L ammonium acetate in acetonitrile and mobile phase B consisted of 0.1% formic acid and 10 mmol/L ammonium acetate in water. The column temperature was set to 40°C. The flow rate was 300 μL/min and the injection volume was 1 μL. The gradient conditions for HILIC separation of polar molecule are shown in Table S2.

Identification of metabolites was performed on a Thermo Scientific™ Q Exactive™ hybrid quadrupole Orbitrap mass spectrometer equipped with a HESI-II probe, which was connected to the RSLC system. Both sheath gas and auxiliary gas were nitrogen. The capillary voltage was set to 3,700 V and the capillary temperature was 320°C. The sheath gas pressure was 30 psi, the auxiliary gas setting was 10 psi, and the heated vaporizer temperature was 300°C. The sheath gas flow and auxiliary gas flow were 30 and 8 L/h respectively. The mass to charge ratio (*m/z*) of the quadrupole's scan ranged from 100 to 1,500. The tandem mass spectrometry data were collected with the collision energy between 10 and 35 eV. Argon at a pressure of 1.5 mTorr was used as collision gas. The parameters of the full mass scan were set as follows: a resolution of 70,000, an auto gain control target under 1 × 10^6^, and a maximum isolation time of 50 ms.

To ensure the data quality of metabolic profiling, pooled quality control (QC) samples were prepared by mixing all of the samples. In our study, eight QC samples were prepared and the pretreatment of QC samples was performed according to the aforementioned protocols. Before analyzing the sample sequence, three QC samples were run. During analysis of the sample sequence, one QC sample was run after every six injections.

### Targeted metabolomics analyses

The candidate SCMs were identified in Discovery phase based on non-targeted metabolomics analyses. In order to validate the abundance of candidate SCMs in Validation phase, hydrophilic interaction liquid chromatography and polar reversed phase chromatography analyses based targeted metabolomics experiments were performed, respectively.

### Data processing and analysis

The UPLC-MS raw data were analyzed by progenesis QI software (Waters Corporation, Mifold, MA, USA) using the following parameters: sensitivity of the picking algorithm was automatic and the sensitivity value was default, chromatographic peak width was 0.05 min, retention time range was 0.7–14 min. Duplicated peaks were excluded from the analysis. Finally, the Excel file including detailed data of *m/z*, retention time and peak intensities of each ion was obtained and underwent further filtration. QC data were used for internal normalization. Peaks were ruled out based on the following criteria: (1) peak area <2,000,000 through the C_18_ column; (2) peak area <10,000 through the HILIC column; Furthermore, ions identified as electrospray ionization positive (ESI^+^) with statistical significance were subjected to multivariate analysis to visualize the metabolic alteration among groups, including principal component analysis (PCA) and orthogonal partial least-squares-discriminant analysis (OPLS-DA), which were applied with unit variance scaling. PCA and OPLS-DA used the unsupervised and supervised methods for pattern recognition, respectively. PCA models and OPLS-DA models were constructed by using EZinfo 3.0 software (Waters Corporation, Mifold, MA, USA) and the corresponding parameters of models including R2X, R2Y, and Q2 were obtained, which were used to ensure he quality of the multivariate models and to avoid the risk of over-fitting. R2X value (range: 0–1) indicates the goodness of fit express how well the model fits the data and Q2 value (range: 0–1) indicates the goodness of prediction express how well the model predicts new data). The variable importance in the projection (VIP) value was generated from OPLS-DA models. Those ions with VIP > 1 and *P* < 0.05 were considered as different metabolites in our work. Hierarchical cluster analysis was conducted using the pheatmap package in R language.

### Confirmation of ions

Tandem mass spectrometry was used to identify the metabolites. The *m/z* and molecular mass of ions was aligned with the Human Metabolome Database (HMDB, http://www.hmdb.ca/). In addition, the fragmentation patterns of metabolites were compared with the fragmentation pattern of the standard and parent ions in the HMDB database.

### Pathway enrichment analysis

In order to predict the biological roles of identified metabolites in MM, MetaboAnalyst 4.0 (http://www.metaboanalyst.ca/faces/ModuleView.xhtml) was used for pathway enrichment analysis.

### Statistical analysis

We used SPSS22.0 software (IBM Corp., Armonk, NY, USA) statistical software for data analysis, Student's *t*-test and receiver operating characteristic (ROC) curves were used in this study. The cutoff value of abundance of SCM, calculated based on ROC curve and Youden index (Luo and Xiong, 2013; Bantis et al., 2014), was used to group MM patients into higher SCM group with more than cutoff value and lower SCM group with less than cutoff value. In detail, in ROC analysis, the diagnostic index including sensitivity and 1-specificity of metabolites in each of subjects in AG and RG groups of Cohort 2, was calculated by SPSS22.0 software. In order to find the optimal diagnostic index, the Youden index was calculated according to the formula: Youden index = sensitivity-(1-specificity). The metabolite value of max Youden index was defined as the cutoff value. The statistical difference of survival period between higher SCM group and lower SCM group was accessed through Kaplan-Meier analysis. Those SCMs and clinical features were used for Cox regression analysis to identify the statistically significantly risk factor of survival period in MM patients.The correlation analysis between 12 SCMs and IgG M protein was accessed through SPSS software. *P* < 0.05 was considered as a significant difference. ^*^ indicates *P* < 0.05; ^**^ indicates *P* < 0.01 and ^***^ indicates *P* < 0.001.

## Results

### Patient demographics

A total of 36 subjects were enrolled in Discovery phase. The mean age of subjects in the RG, AG and HC groups was respectively 57.9, 63.5, and 63.3 years. RG group included six males and four females, AG group included eight males and four females, and HC group included six males and eight females. A total of 75 subjects were enrolled in Validation phase. The mean age of subjects in the RG, AG, and HC groups was respectively 61.7, 61.3, and 64.7 years. RG group included 16 males and 10 females, AG group included 17 males and 12 females and HC group included 8 males and 12 females. There was no significant difference in the age or gender among the RG, AG, and HC in Discovery phase and Validation phase (*P* > 0.05).

More than 40% of MM patients were IgG type, and more than 80% of patients had entered clinical phase II and III when diagnosed (Tables 1, 2). As shown in Tables 1, 2, the clinical features including age, gender, type of MM stage of R-ISS between AG and RG groups were not statistically significant both in Cohort 1 and Cohort 2, moreover, the clinical feature of plasma cell, Hemoglobin, β_2−_MG > 5.5 mg/dl, Creatinine, Globulin, amount of immunoglobulin G (IgG) between the AG and RG groups were significantly different (*P* < 0.05) both in Cohort 1 and Cohort 2.

### Metabolic profiling of serum samples in the AG, RG, and HC groups in discovery phase

In our study, a non-targeted metabolomics strategy was applied in the Discovery phase. Liquid chromatography was used to separate lipid and polarized components in serum samples. Typical UPLC-MS total ion chromatograms of serum samples on a C_18_ column and a HILIC column under ESI positive (ESI^+^) mode for AG, RG and HC groups are shown in Figures S1A,B. The metabolic profiles of the AG and RG groups were substantially different from that of the HC group both in the C_18_ column and the HILIC column under ESI^+^ mode. In addition, a substantial difference was also observed between the AG and RG groups.

### PCA and OPLS-DA

**Figure 2:**
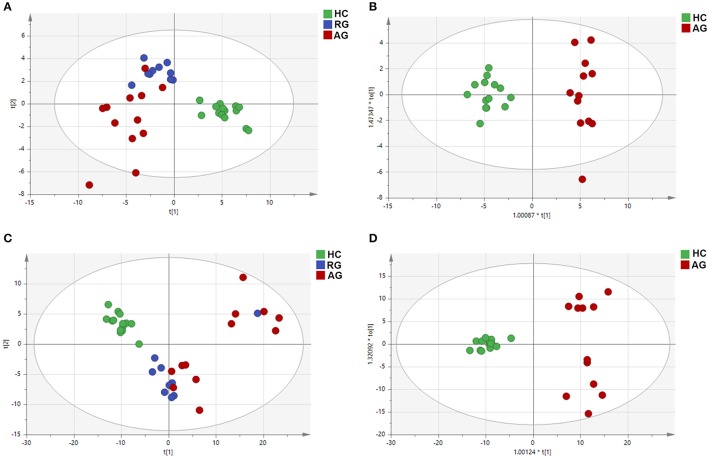
PCA and OPLS-DA of the metabolic profiles of serum samples in HC, RG, and AG groups of Discovery phase analyzed by C_18_ and HILIC column chromatography. **(A)**, PCA analysis in C_18_ mode; **(B)**, OPLS-DA analysis in C_18_ mode; **(C)**, PCA analysis in HILIC mode; **(D)**, OPLS-DA analysis in HILIC mode. Red nodes, blue nodes and green nodes respectively represented the samples of the subjects in AG, RG, and HC groups. PCA: principal components analysis; OPLS-DA: orthogonal partial least-squares-discriminant analysis; HILIC: hydrophilic interaction liquid chromatography. The HC, RG, and AG groups in Discovery phase included 14, 10, 12 subjects, respectively.

PCA, using the unsupervised model, was performed to reveal differences in the metabolic profiling of samples among groups. The PCA score plot exhibited clear clusters of serum samples among the AG, RG and HC groups both in the C_18_ column (*R*^2^*X* = 0.761, *Q*^2^ = 0.604) and the HILIC column (*R*^2^*X* = 0.780, *Q*^2^ = 0.624) under ESI^+^ modes, as shown in Figures 2A,C. For further analysis of the metabolic differences among the AG and HC groups, OPLS-DA, a supervised method for pattern recognition, was applied. As illustrated by the OPLS-DA score plot, serum samples in AG group were clearly separated from those in HC group both in the C_18_ column (*R*^2^*X* = 0.745, *Q*^2^ = 0.644) and the HILIC column (*R*^2^*X* = 0.628, *Q*^2^ = 0.560) mode (Figures 2B,D).

### Identification of differential metabolites in AG compared with HC in discovery phase based on C18 column chromatography

OPLS-DA score plots were used to identify differential metabolites for distinguishing AG and RG from HC group. A total of 20 ions with a VIP > 1 and *P* < 0.05, was considered as significantly changed metabolites (SCMs), were selected for subsequent chemical structure identification, in the C_18_ column under ESI^+^ mode. The 20 metabolites identified are presented in Table S3.

**Figure 3:**
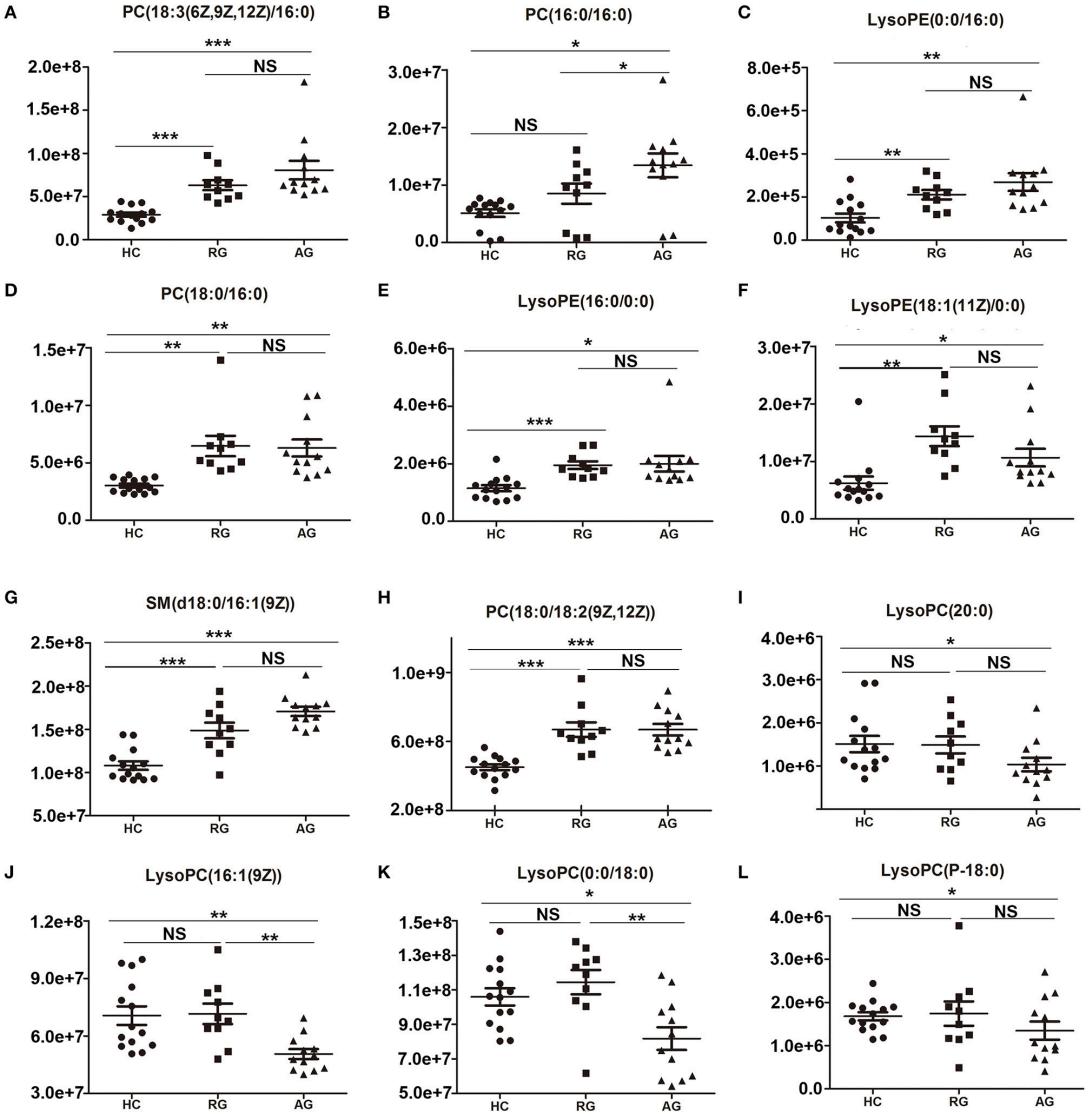
Histogram showing the abundance of SCMs generated from C_18_ column chromatography in AG, RG, and HC groups of Discovery phase. **(A)**, PC(18:3(6Z,9Z,12Z)/16:0); **(B)**, PC(16:0/16:0); **(C)**, LysoPE(0:0/16:0); **(D)**, PC(18:0/16:0); **(E)**, LysoPE(16:0/0:0); **(F)**, LysoPE(18:1(11Z)/0:0); **(G)**, SM(d18:0/16:1(9Z)); **(H)**, PC(18:0/18:2(9Z,12Z)); **(I)**, LysoPC(20:0)); **(J)**, LysoPC(16:1(9Z)); **(K)**, LysoPC(0:0/18:0); **(L)**, LysoPC(P-18:0). *indicates *P* < 0.05, **indicates *P* < 0.01, and ***indicates *P* < 0.001. *P* < 0.05 indicates statistical significance. SCMs, significantly changed metabolites. The HC, RG, and AG groups in Discovery phase included 14, 10, 12 subjects, respectively.

The abundance of those 20 SCMs in HC, RG and AG groups were displayed in Figure 3 and Figure S2. PC(18:3(6Z,9Z,12Z/16:0)), PC(16:0/16:0), LysoPE(0:0/16:0) and PC(18:0/16:0), the top 4 identified SCMs based on fold change, were significantly elevated in AG compared with HC (Figures 3A–D); moreover, PC(18:3(6Z,9Z,12Z/16:0)), LysoPE(0:0/16:0) and PC(18:0/16:0) were also significantly elevated in RG group compared with HC group, in addition, there was none of significance of PC(16:0/16:0) between RG and HC groups. As Figures 3E–H shown, LysoPE(16:0/0:0), LysoPE(18:1(11Z)/0:0), SM(d18:0/16:1(9Z)) and PC(18:0/18:2(9Z,12Z)) were significantly elevated both in RG and AG compared with HC. LysoPC(20:0), LysoPC(16:1(9Z)), LysoPC(0:0/18:0) and LysoPC(P-18:0) were significantly decreased in AG compared in HC and had none of significant change in RG compared with HC, as Figures 3I–L shown.

### Identification of differential metabolites in AG compared with HC in discovery phase based on HILIC column chromatography

In HILIC column under ESI^+^ mode, 22 SCMs were identified in AG group compared with HC group according to the cutoff of VIP > 1 and *P* < 0.05 based on OPLS-DA model analysis (Table S4). 2-Hexenoylcarnitine, 1-Methylhistidine and Asymmetric dimethylarginine, with respective fold change of 7.898, 7.578, and 6.632, were the top 3 SMCs in AG group compared with HC group, as Table S4 shown.

**Figure 4:**
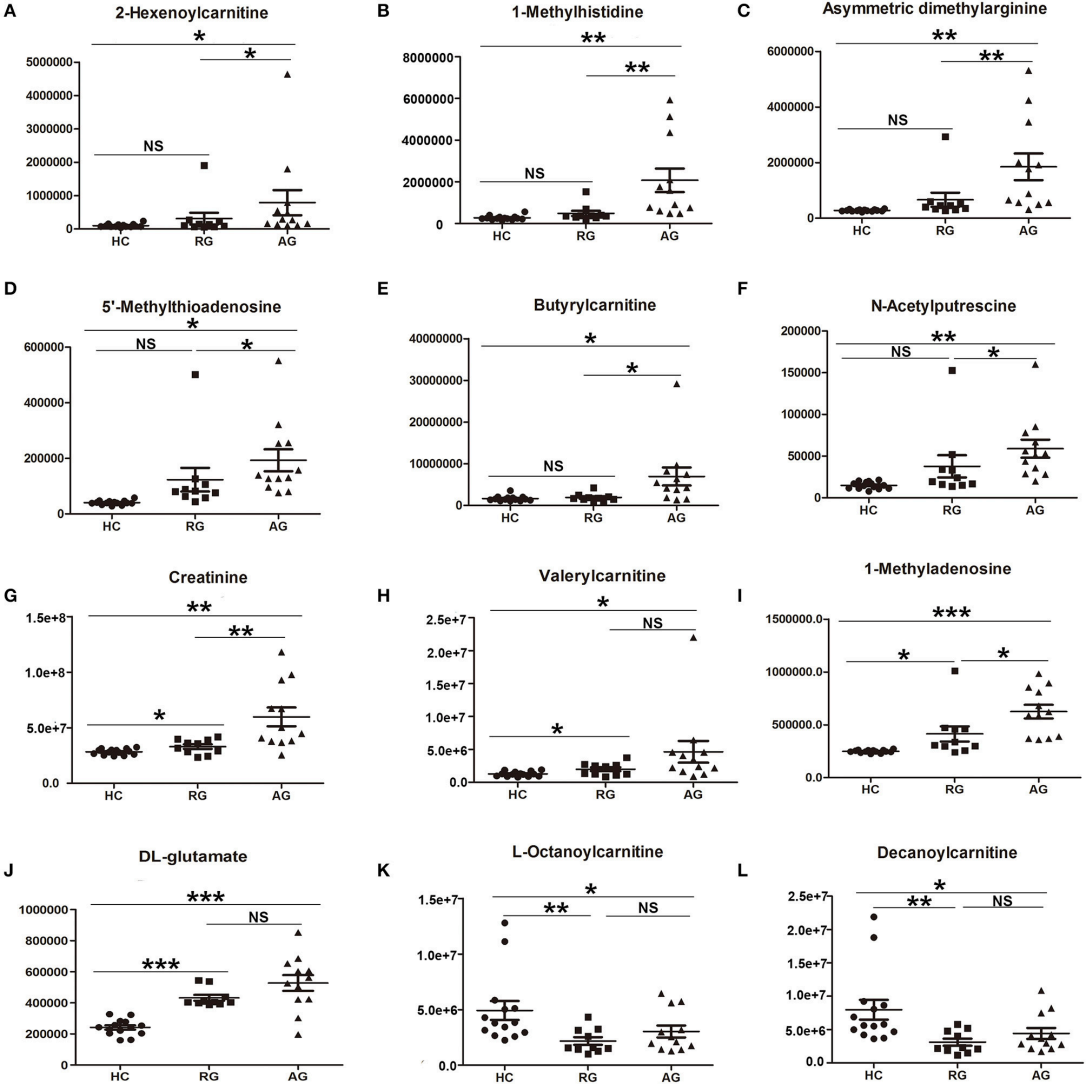
Histogram showing the abundance of SCMs generated from HILIC column chromatography in AG, RG, and HC groups of Discovery phase. **(A)**, 2-Hexenoylcarnitine; **(B)**, 1-Methylhistidine; **(C)**, Asymmetric dimethylarginine; **(D)**, 5′-Methylthioadenosine; **(E)**, Butyrylcarnitine; **(F)**, N-Acetylputrescine; **(G)**, Creatinine; **(H)**, Valerylcarnitine; **(I)**, 1-Methyladenosine; **(J)**, DL-glutamate; **(K)**, L-Octanoylcarnitine; **(L)**, Decanoylcarnitine. *indicates *P* < 0.05, **indicates *P* < 0.01, and ***indicates *P* < 0.001. *P* < 0.05 indicates statistical significance. SCMs, significantly changed metabolites. The HC, RG, and AG groups in Discovery phase included 14, 10, 12 subjects, respectively.

The abundance of those 22 SCMs in HC, RG and AG groups were displayed in Figure 4 and Figure S3. As Figures 4A–J shown, 2-Hexenoylcarnitine, 1-Methylhistidine, Asymmetric dimethylarginine, 5′-Methylthioadenosine, Butyrylcarnitine, N-Acetylputrescine, Creatinine, Valerylcarnitine, 1-Methyladenosine, DL-Glutamate were all significantly elevated in AG compared with HC. Moreover, L-Octanoylcarnitine (Figure 4K) and Decanoylcarnitine (Figure 4L) were significantly decreased in AG compared with HC. In addition, Creatinine (Figure 4G), Valerylcarnitine (Figure 4H), 1-Methyladenosine (Figure 4I), and DL-Glutamate (Figure 4J), were also significantly elevated in RG compared with HC, and L-Octanoylcarnitine (Figure 4K) and Decanoylcarnitine (Figure 4L) was also significantly decreased in RG compared with HC.

### Validation of the abundance of identified SCMs in validation phase

**Figure 5:**
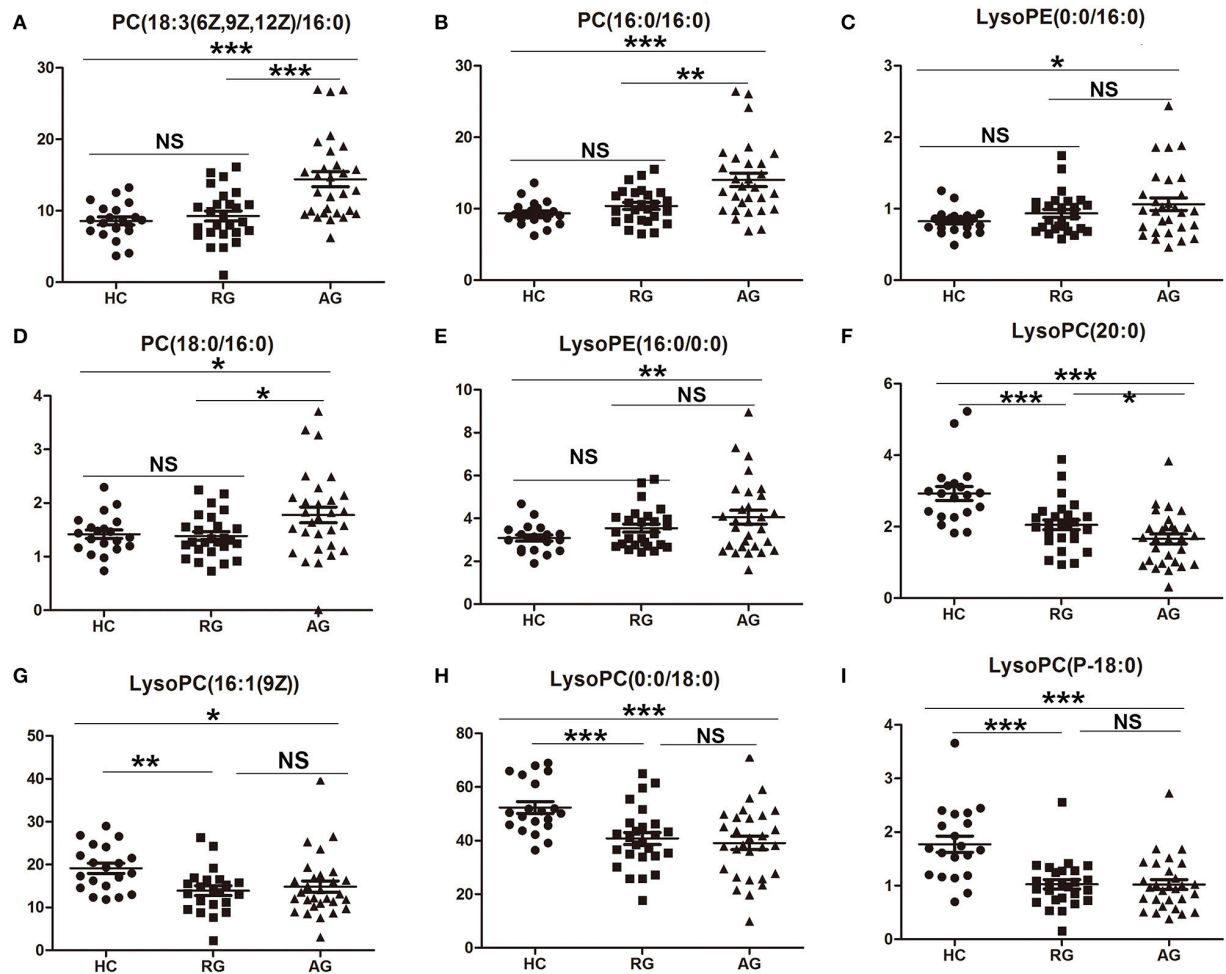
Validation of the abundance of SCMs generated from C_18_ column chromatography in AG, RG, and HC groups in Validation phase. **(A)**, PC(18:3(6Z,9Z,12Z)/16:0); **(B)**, PC(16:0/16:0); **(C)**, LysoPE(0:0/16:0); **(D)**, PC(18:0/16:0); **(E)**, LysoPE(16:0/0:0); **(F)**, LysoPC(20:0)); **(G)**, LysoPC(16:1(9Z)); **(H)**, LysoPC(0:0/18:0); **(I)**, LysoPC(P-18:0). *indicates *P* < 0.05, **indicates *P* < 0.01, and ***indicates *P* < 0.001. *P* < 0.05 indicates statistical significance. SCMs, significantly changed metabolites. Targeted metabolomics analyses was used to validate the abundance of SCMs in Validation phsge. The HC, RG, and AG groups in Validation phase included 20, 26, 29 subjects, respectively.

Total of 42 SCMs had been identified in AG group compared with HC group in Discovery phase based on C18 column and HILIC column under ESI^+^ model. In order to validate the abundance of the SCMs, targeted metabolomics analysis was performed and the concentration of SCMs was calculated in Validation phase. The difference of the abundance of SCMs among HC, RG, and AG groups in Validation phase had been analyzed. The abundance of 20 identified SCMs based on C18 Column in Discovery phase was validated in Validation phase. 10 out of 20 SCMs was significantly changed in AG group compared with HC group in Validation phase. Except for PC(18:0/18:2(9Z,12Z)) (Figure S4), the abundance of 9 SCMs identified in Discovery phase had the same tendency in Validation phase. As Figure 5 shown, PC(18:3(6Z,9Z,12Z/16:0)) (Figure 5A), PC(16:0/16:0) (Figure 5B), LysoPE(0:0/16:0) (Figure 5C), PC(18:0/16:0) (Figure 5D), and LysoPE(16:0/0:0) (Figure 5E) were significantly elevated in AG compared with HC in Validation phase. As Figures 5F–I shown, LysoPC(20:0), LysoPC(16:1(9Z)), LysoPC(0:0/18:0), and LysoPC(P-18:0) were significantly decreased in AG compared with HC in Validation phase.

**Figure 6:**
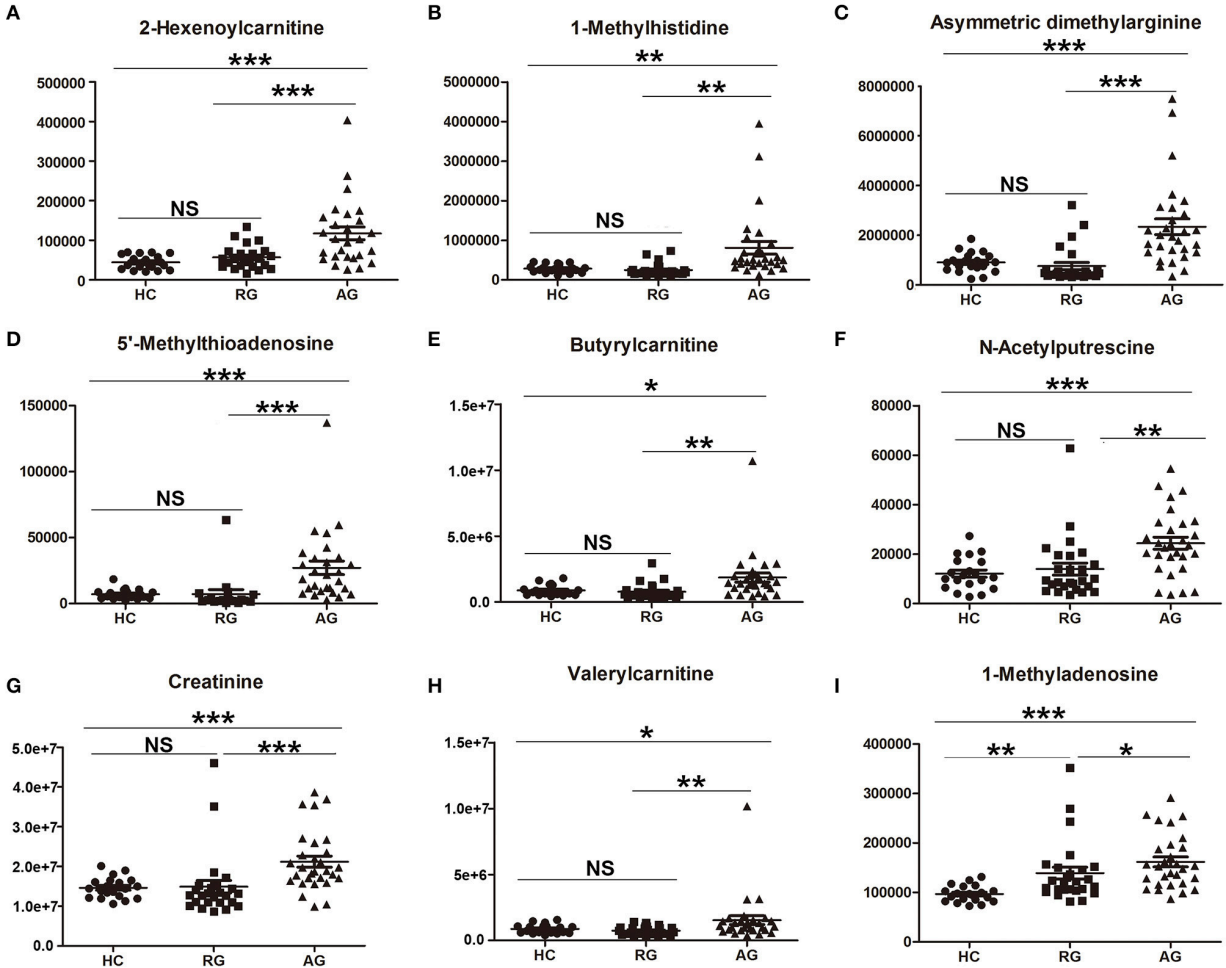
Validation of the abundance of SCMs generated from HILIC column chromatography in AG, RG, and HC groups in Validation phase. **(A)**, 2-Hexenoylcarnitine; **(B)**,1-Methylhistidine; **(C)**, Asymmetric dimethylarginine; **(D)**, 5′-Methylthioadenosine; **(E)**, Butyrylcarnitine; **(F)**, N-Acetylputrescine; **(G)**, Creatinine; **(H)**, Valerylcarnitine; **(I)**, 1-Methyladenosine. *indicates *P* < 0.05, **indicates *P* < 0.01, and ***indicates *P* < 0.001. *P* < 0.05 indicates statistical significance. SCMs, significantly changed metabolites. Targeted metabolomics analyses was used to validate the abundance of SCMs in Validation phase. The HC, RG, and AG groups in Validation phase included 20, 26, 29 subjects, respectively.

In addition, the abundance of 22 identified SCMs based on HILIC Column in Discovery phase was also validated in Validation phase. 14 out of 22 SCMs was significantly changed in AG compared with HC in Validation phase (Figure 6 and Figure S5). The abundance of all of 14 SCMs identified in Discovery phase had the same tendency in Validation phase. As Figures 6A–I shown, 2-Hexenoylcarnitine, 1-Methylhistidine, Asymmetric dimethylarginine, 5'-Methylthioadenosine, Butyrylcarnitine, N-Acetylputrescine, Creatinine, Valerylcarnitine and 1-Methyladenosine were all significantly elevated in AG compared with HC in Validation phase. As Figure S5 shown, DL-Glutamate (Figure S5A), 3-Dehydroxycarnitine (Figure S5D) and L-Proline (Figure S5E) were also significantly elevated in AG compared with HC, and L-Octanoylcarnitine (Figure S5B) and Decanoylcarnitine (Figure S5C) were significantly decreased in AG compared with HC in Validation phase.

In summary, the abundance of 23 SCMs in AG group compared with HC group in Discovery phase had the same tendency in Validation phase. 14 SCM was significantly elevated in AG compared with HC both in Discovery phase and Validation phase, and 9 SCMs was significantly decreased in AG compared with HC both in Discovery phase and Validation phase.

### Investigation of the diagnostic potential of metabolites between MM patients and HC in validation phase

**Figure 7:**
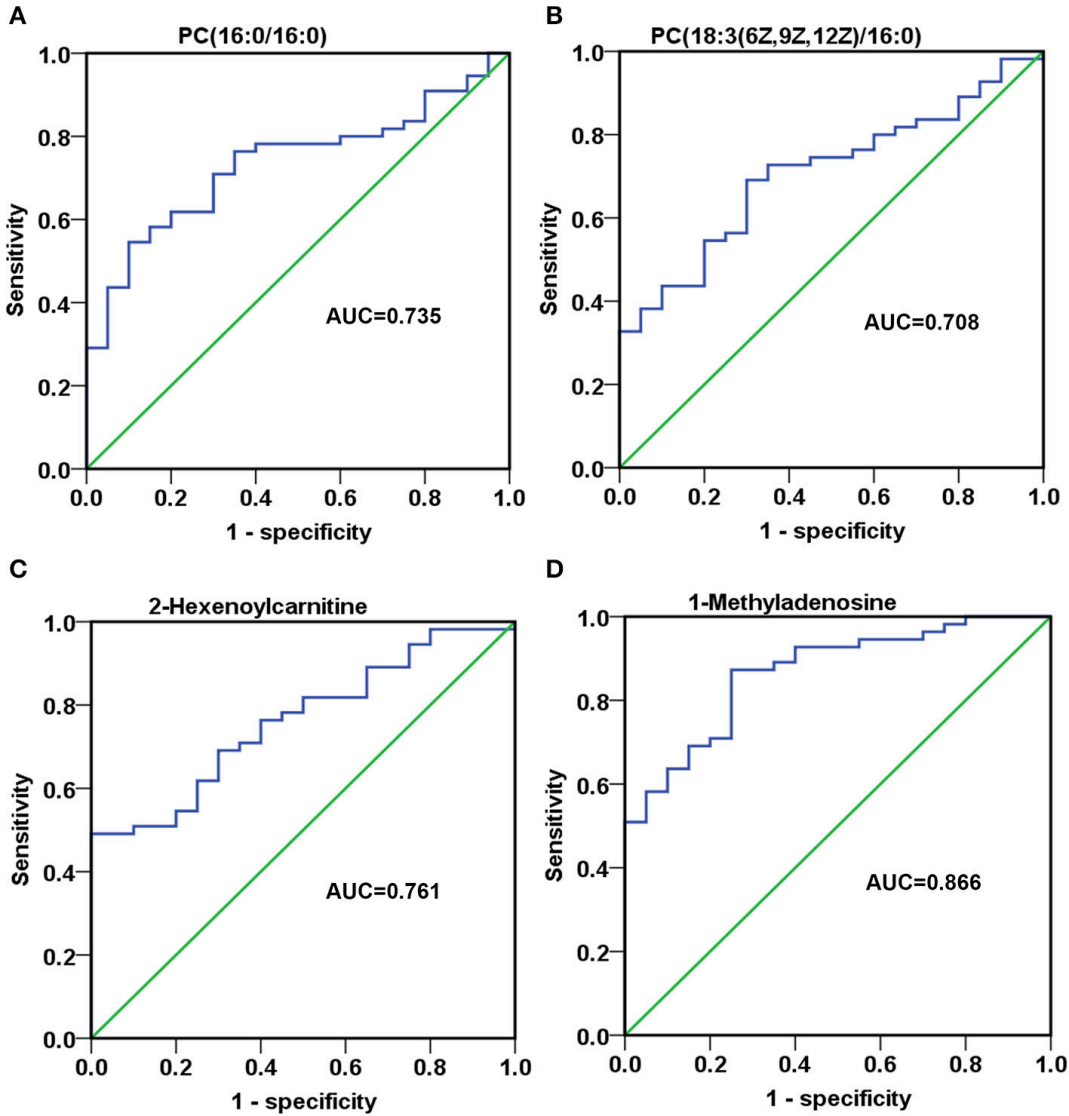
The discriminatory ability of SCMs between MM patients and healthy controls was analyzed using an ROC curve in Validation phase. **(A)**, PC(16:0/16:0); **(B)**, PC(18:3(6Z,9Z,12Z)/16:0); **(C)**, 2-Hexenoylcarnitine; **(D)**, 1-Methyladenosine. ROC, receiver operating characteristic curve. ROC curves and AUC were obtained by SPSS22.0 software. The HC, RG, and AG groups in Validation phase included 20, 26, 29 subjects, respectively.

To assess the discriminatory ability of the 23 aforementioned SCMs between MM patients and healthy controls, ROC analyses were applied for calculation of the area under the curve (AUC). 4 out of 23 SCMs had the diagnostic value in distinguishing the plasma samples of MM patients from those of healthy controls. As shown in Figures 7A–C, the AUC of PC(16:0/16:0), PC(18:3(6Z,9Z,12Z)/16:0) and 2-Hexenoylcarnitine was 0.735, 0.708, and 0.761, respectively. The AUC of 1-Methyladenosine was 0.866 (Figure 7D), which could obviously distinguish MM patients from healthy controls.

### Investigation of the diagnostic potential of metabolites among AG and RG in validation phase

**Figure 8:**
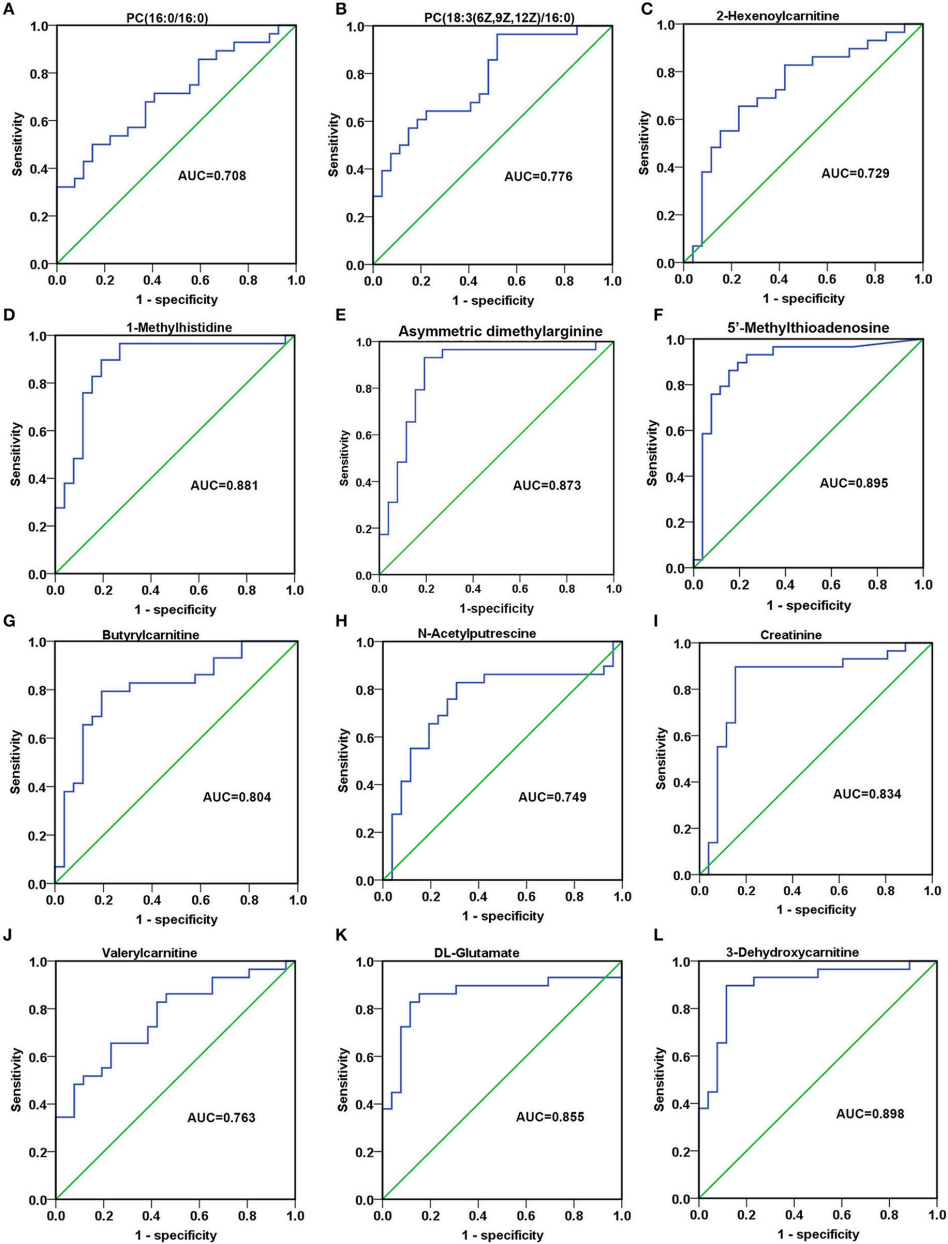
The discriminatory ability of SCMs between AG patients and RG patients was analyzed using an ROC curve in Validation phase. **(A)**, PC(16:0/16:0); **(B)**, PC(18:3(6Z,9Z,12Z)/16:0); **(C)**, 2-Hexenoylcarnitine; **(D)**, 1-Methylhistidine; **(E)**, Asymmetric dimethylarginine; **(F)**, 5′-Methylthioadenosine; **(G)**, Butyrylcarnitine; **(H)**, N-Acetylputrescine; **(I)**, Creatinine; **(J)**, Valerylcarnitine; **(K)**, DL-Glutamate; **(L)**, 3-Dehydroxycarnitine. ROC, receiver operating characteristic curve. ROC curves and AUC were obtained by SPSS22.0 software. The HC, RG, and AG groups in Validation phase included 20, 26, 29 subjects, respectively.

In order to detect the discriminatory ability of those identified 23 SCMs among AG patients and RG patients, the ROC analyses were performed. Finally, 12 out of 23 SCMs had the diagnostic value in distinguishing plasma samples of AG patients from RG patients. As Figures 8A,B shown, the AUC of PC(16:0/16:0) and PC(18:3(6Z,9Z,12Z)/16:0) was 0.708 and 0.776, respectively. The AUC of 2-Hexenoylcarnitine (Figure 8C), N-Acetylputrescine (Figure 8H) and Valerylcarnitine (Figure 8J) was more than 0.7. The AUC of 1-Methylhistidine (Figure 8D), Asymmetric dimethylarginine (Figure 8E), 5′-Methylthioadnosine (Figure 8F), Butyrylcarnitine (Figure 8G), Creatinine (Figure 8I), DL-Glutamate (Figure 8K) and 3-Dehydroxycarnitine (Figure 8L) was more than 0.8. In addition, the AUC of 3-Dehydroxycarnitine (Figure 8L), 5′-Methylthioadnosine (Figure 8F) and 1-Methyhistidine (Figure 8D) was respective 0.898, 0.895 and 0.881; those 3 SCMs had the high diagnostic value in distinguishing AG patients from RG patients.

### Reconfirm the clinical significance of SCMs

For the type of IgG MM patients, IgG M protein is an important clinical indicator for judging the progression or remission of MM. Therefore, the correlation analyses between the serum level of M protein and 12 SCMs were performed. As Table 3 shown, except for 1-Methyladenosine and PC (18:3(6Z,9Z,12Z)/16:0), 10 SCMs were positively correlated with IgG M protein.

**Table 3:** The correlation analysis between SCMs and IgG M protein in MM patients in Cohort 2.

**No**.	**SCM**	***r***	***P*-value**
1	Butyrylcarnitine	0.705	0.000
2	Valerylcarnitine	0.439	0.036
3	2-Hexenoylcarnitine	0.518	0.000
4	N-Acetylornithine	0.608	0.002
5	Asymmetric dimethylarginine	0.513	0.009
6	3-Dehydroxycarnitine	0.548	0.007
7	5'-Methylthioadenosine	0.568	0.005
8	Creatinine	0.445	0.033
9	PC(16:0/16:0)	0.417	0.048
10	DL-Glutamate	0.431	0.040
11	1-Methyladenosine	0.053	0.811
12	PC(18:3(6Z,9Z,12Z)/16:0)	0.355	0.118

*No., number; SCM, significantly changed metabolite*.

**Figure 9:**
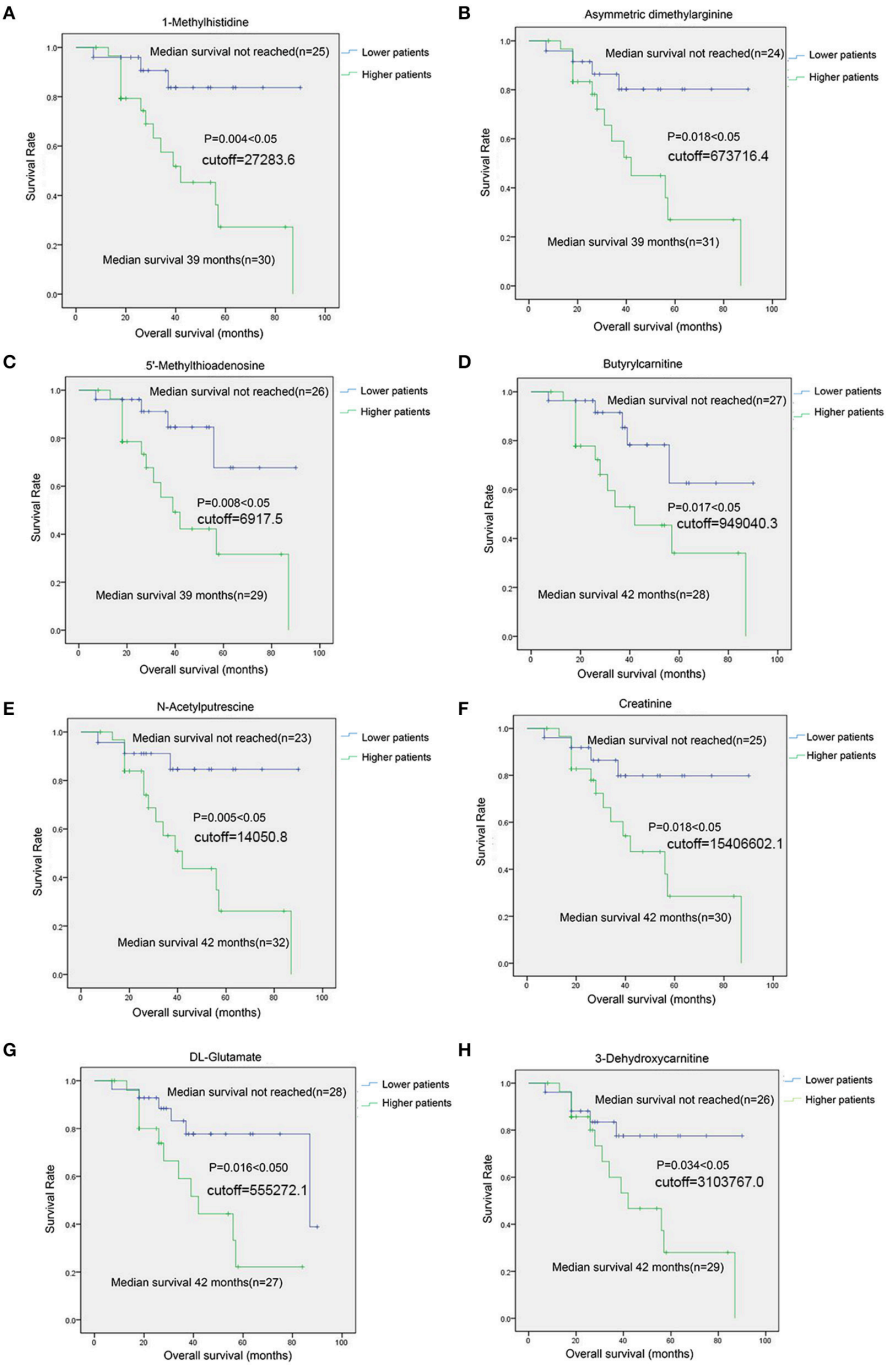
The survival analysis of identified SCMs in MM patients in Cohort 2. **(A)**, 1-Methylhistidine; **(B)**, Asymmetric dimethylarginine; **(C)**, 5′-Methylthioadenosine; **(D)**, Butyrylcarnitine; **(E)**, N-Acetylputrescine; **(F)**, Creatinine; **(G)**, DL-Glutamate; **(H)**, 3-Dehydroxycarnitine. Twenty-six Subjects of RG group and 29 subjects of AG groups in Cohort 2 was conduted to survival analyses. The cutoff value was calculated by ROC analysis and Youden index. Those 55 subjects were grouped into lower patients group and higher patients group based on the cutoff value. Patients with metabolite value less than cutoff value was incorporated into lower patient goup and those patients with metabolite value more than cutoff vuale was incorporated into higher paitent group, respectively. There was none of unit for the cutoff values of metabolites shown in Figure on account of the standard substance of metabolites identified in HILIC column is absent and we could not obtain the concentration of each of metabolites shown in Figure. The metabolites shown in Figure in each of samples was quantitative by the peak area generated from mass spectrometer.

For 8 SCMs of 3-Dehydroxycarnitine, DL-Glutamate, Creatinine, N-Acetylputrescine, Butyrylcarnitine, 5′-Methylthioadenosine, Asymmetric dimethylarginine and 1-Methylhistidine, the survival analysis indicated that MM patients with higher level SCMs had the shorten overall survival(OS) compared with those patients with lower level of SCMs (Figure 9).

In addition, the Cox regression model analysis found that age and only 5′-Methylthioadenosine in 12 SCMs was the independent prognostic factors in survival period of MM patients (Table 4).

**Table 4:** Cox regression analysis of multivariate in survival period of MM patients in Cohort 2.

**Variates**	**Wald**	***P*-value**	**OR**	**95% CI**
Age	5.472	0.019	0.869	0.772–0.977
Gender	0.516	0.473	2.170	0.262–17.975
Hemoglobin	2.131	0.144	0.119	0.007–2.074
Creatinine	1.524	0.217	5.374	0.372–77.628
β_2_-MG	0.688	0.407	2.139	0.355–12.903
Globulin	3.067	0.080	0.132	0.014–1.272
LDH	1.378	0.240	0.213	0.016–2.816
PC(18:3(6Z,9Z,12Z)/16:0)	0.620	0.431	3.571	0.150–84.843
PC(16:0/16:0)	0.303	0.582	1.713	0.252-11.622
3-Dehydroxycarnitine	1.170	0.279	0.080	0.001–7.746
DL-Glutamate	0.030	0.862	1.283	0.077–21.294
Valerylcarnitine	0.152	0.697	0.593	0.043–8.227
Creatinine	2.311	0.128	38.664	0.348–4,301.003
N-Acetylputrescine	3.695	0.055	36.652	0.932–1,441.811
Butyrylcarnitine	3.482	0.062	39.725	0.831–1,899.498
5′-Methylthioadenosine	4.408	0.036	0.000	0.000–0.595
Asymmetric dimethylarginine	1.579	0.209	0.015	0.000–10.571
1-Methylhistidine	1.333	0.248	23.191	0.112–4,822.052
2-Hexenoylcarnitine	0.457	0.499	0.332	0.014–8.126

*OR, odds ration; P < 0.05 is statistical significance; β_2_-MG, β_2_-microglobulin; LDH, lactate dehydrogenase*.

### The potential biological functions of SCMs in MM

**Figure 10:**
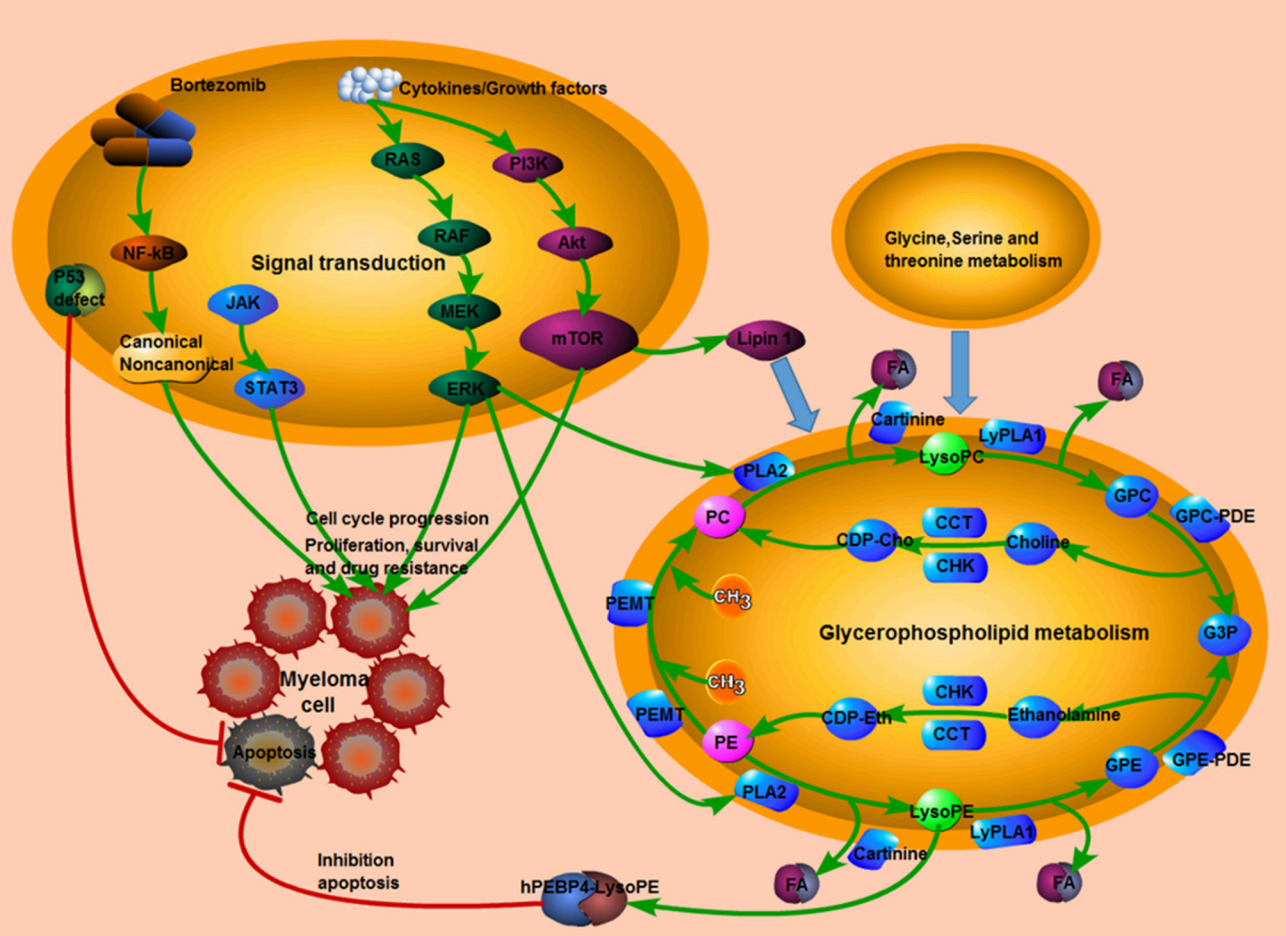
The tumorigenesis model in MM. The interaction between reported signaling pathways and potential metabolic signaling pathways identified in our work which might be implicated in the pathogenesis of MM. Pink indicated elevated metabolites in the study; green indicated decreased metabolites in the study; red line indicated an inhibitory effect. MM, multiple myeloma.

MetaboAnalyst 4.0 software was conduct to understand the pathways involved in those 23 identified SCMs. In summary, two metabolic pathways, arginine and proline metabolism and glycerophospholipid metabolism, were significantly enriched (Table S5). L-Proline, Creatinine and N-Acetylputrescine were significantly enriched in Arginine and proline metabolism pathway; LysoPC(18:1(9Z)) and Phosphatidylcholine (including PC(16:0/16:0), PC(18:3(6Z,9Z,12Z)/16:0) and PC(18:0/16:0)) were significantly enriched in Glycerophospholipid metabolism pathway. Moreover, the reported signaling pathways involved in cell growth, cell proliferation, cell cycle, drug resistance in MM and the potential metabolic signaling pathways identified in our work were schematically depicted in Figure 10, which shown the MM tumorigenesis model at both the metabolic level and the cellular level.

## Discussion

Lipids are vital signaling molecules regulating biological processes under normal and diseased conditions. Mounting evidence demonstrates that the altered metabolites are correlated with the development and treatment of cancer. In the chemoradiotherapy of rectal cancer, the levels of five serum metabolites including PC40, SM(d18:2/18:1), LysoPC(16:0/0:0), LysoPC(15:1(9Z)/0:0), and LysoPE(22:5/0:0) are significantly higher in responders than in non-responders, and this five-lipid signature has been shown to possess sensitivity and specificity in predicting the tumor response to chemoradiotherapy (Del Boccio et al., 2017).

Medriano et al. found that pyrimidine metabolism, carbon metabolism, and bile secretion pathways were potentially affected by MM (Manier et al., 2017), while Lodi et al. identified carnitine and acetylcarnitine as novel potential biomarkers of active disease both at diagnosis and relapse in MM (Lodi et al., 2013), and Jones et al. reported the metabolomics profiling of bortezomib-sensitive and bortezomib-resistant MM cells were obviously distinct (Jones et al., 2014).

Studies have reported the metabolomics profiling of human MM (Lodi et al., 2013; Jones et al., 2014; Medriano et al., 2017), but the global metabolomics of MM in Chinese patients is ambiguous. Furthermore, published studies describing the MM metabolome focus on a relatively small sample size (three patients with MM and six healthy individuals) or compare drug-sensitive/drug resistant MM cell lines (Jones et al., 2014; Medriano et al., 2017). To address this hiatus, the global metabolomics of serum samples from different disease status of MM patients were deciphered in our analyses, including active MM patients, responding after treatment and normal controls.

In our analyses, the abundance of PC(16:0/16:0), PC(18:0/16:0) and PC(18:3(6Z,9Z,12Z)/16:0) were all significantly elevated both in AG group compared with HC group both in Discovery phase and Validation phase. The molecular formula of PC(16:0/16:0), PC(18:0/16:0), and PC(20:0/18:3(6Z,9Z,12Z)) are C_40_H_80_NO_8_P, C_42_H_84_NO_8_P and C_42_H_78_NO_8_P, respectively. These three SCMs belong to the phosphatidylcholines (PC or GPCho)/lecithins. Phospholipids are ubiquitous in nature and are key components of the lipid bilayer of cells, as well as being involved in metabolism and signaling.

A series of studies have reported that the combination of PC(16:0/16:0) and an anti-cancer agent results in fast treatment efficacy in treatment of lung cancer, breast cancer and colon cancer (Alavizadeh et al., 2017; Tagami et al., 2017; Tran et al., 2017). Also, the abundance of phosphatidylcholines is commonly altered in various tumors. PC(16:0/16:0), also known as dipalmitoylphosphatidylcholine (DPPC), colfosceril palmitate and PC (32:0), contains two chains of palmitic acid, which is the major constituent of pulmonary surfactant. DPPC used as surfactant adjunctive therapy improved lung function of an infant with acute lymphoblastic leukemia (Slater et al., 1995). Furthermore, along with PC34:1, PC36:2, DPPC is more abundant in HER2-positive breast cancer compared to adjacent normal tissues (Kim et al., 2013). In non-small cell lung cancer (NSCLC), the relative abundance of DPPC is obviously lower and [DPPC+H]+, [DPPC+Na]+, and [DPPC+K]+ are more abundant in the sputum of NSCLC patients than in controls; consequently the abundance of [DPPC+H]+, [DPPC+Na]+, and [DPPC+K]+ could distinguish NSCLC patients from controls, which might be a noninvasive and effective biomarker for the diagnosis of NSCLC (Zhang J. et al., 2016). In our work, and the abundance of PC(16:0/16:0) was significantly elevated in AG group compared with HC group and significantly correlated with serum level of IgG M protein, which indicated that PC(16:0/16:0) might be involved in the progressiveness of MM.

PC(18:0/16:0) consists of one chain of stearic acid at the C-1 position and one chain of palmitic acid at the C-2 position. Its official name is 1-stearoyl-2-palmitoyl-sn-glycero-3-phosphocholine. Hypoxic areas are a characteristic of rapidly-growing malignant tumors, which contribute to tumor cell biology and tumor progression. It is reported that PC(18:0/16:0) is observed in hypoxic regions of a breast tumor xenograft model, but not in non-hypoxic areas (Jiang et al., 2015).

LysoPC(20:0), LysoPC(16:1(9Z)), LysoPC(0:0/18:0), and LysoPC(P-18:0), members of the lysophosphatidylcholines (LPCs), which are formed by hydrolysis of phosphatidylcholines (PCs) by the enzyme phospholipase A2, were significantly decreased in AG group compared with HC group both in Discovery phase and Validation phase. LPC is the major bioactive lipid component of oxidized low-density lipoprotein (LDL), thought to be responsible for many of the inflammatory effects of oxidized LDL described in both inflammatory and endothelial cells. LPC has also been found to stimulate basic fibroblast growth factor release as well as stimulating the release of the cytokines granulocyte-macrophage colony-stimulating factor (GM-CSF), interleukin (IL)-6, and IL-8 (Aiyar et al., 2007). Therefore, elevated LPCs may act as inflammatory stimuli. In this study, the release of IL-6 and other inflammatory factors was induced, resulting in the progression of MM.

Renal failure is a common clinical feature of MM patients. Creatinine and asymmetric dimethylarginine (ADMA), were markedly increased in AG group compared with HC group in our study. Creatinine is a breakdown product of creatinine phosphate in muscle, from where it is transferred to the kidneys via the blood plasma. Serum creatinine is the most commonly-used indicator of renal function (Goyal and Bhimji, 2017). A number of articles have reported that serum creatinine is obviously up-regulated in MM patients compared with controls (Umeda et al., 2006), which is consistent with our study. ADMA is an endogenously-produced inhibitor of nitric oxide synthase. However, elevated levels of ADMA occur especially in patients with end-stage chronic kidney disease (CRD). In addition, an increase in plasma ADMA levels of 0.1 μmol/L is associated with a 37% increased risk of death induced by severe illness such as CRD (Goyal and Bhimji, 2017). In our work, serum creatinine and ADMA was significantly positively correlated with the IgG M protein and negatively correlated with the survival period of MM patients.

LysoPE(16:0/0:0) and LysoPE(0:0/16:0) were all significantly increased in AG group compared with HC group both in Discovery phase and Validation phase. The involvement of these lipids in MM may be supported by the known correlation between human phosphatidylethanolamine-binding protein 4 (hPEBP4) and inhibition of apoptosis (Wang et al., 2005; Li et al., 2007).

In our study, the ROC curve analyses indicated that PC(16:0/16:0), PC(18:3(6Z,9Z,12Z)/16:0), 2-Hexenoylcarnitineand and 1-Methyladenosine had high discriminatory ability between MM patients and healthy controls, indicating that they might be potential biomarkers for distinguishing MM patients from healthy individuals. Moreover, PC(16:0/16:0), PC(18:3(6Z,9Z,12Z)/16:0) and 2-Hexenoylcarnitine could be used to discriminate between the AG patients and RG patients. Except for those 3 SCMs abovementioned, 9 SCMs, including 3-Dehydroxycarnitine, 5′-Methylthioadenosine, 1-Methylhistidine, Asymmetric dimethylarginine, DL-Glutamate, Creatinine, Butyrylcarnitine, Valerylcarnitine, and N-Acetylputrescine could discriminate between AG patients and RG patients.

Butyrylcarnitine, Valerylcarnitine and 2-Hexenoylcarnitine, belongs to Acyl carnitines, were significantly elevated in AG patients compared with HC and obviously elevated in AG patients compared with RG patients. It is reported that carnitine and acetylcarnitine are identified as novel biomarkers of active diagnosis and relapse and as a mediator of disease associated pathologies in MM (Lodi et al., 2013). In clinical trials, acetyl-L-carnitine (ALCAR) is used to treat peripheral neuropathy in patients with relapsed MM, however, the desired efficacy was not achieved (Brami et al., 2016). Studies in animal experiments and myeloma cell lines found that carnitine may promote B lymphocytes differentiate into plasma cells and participate in antibody-mediated immune responses by enhancing plasma cell immunoglobulin (Ig) secretion (Athanassakis et al., 2001; Khoo and Al-Rubeai, 2009). In our work, Butyrylcarnitine, Valerylcarnitine and 2-Hexenoylcarnitine were significantly positively correlated with the IgG M protein and Butyrylcarnitine was negatively correlated with the survival period of MM patients. The elevated levels of carnitine might be involved in the pathogenesis of MM when the disease progresses.

5′-Methylthioadenosine (MTA) was significantly up-regulated in AG patients compared with RG patients and could distinguish AG patients from RG patients. Evidence suggests that MTA can affect cellular processes in many ways. For instance, MTA has been shown to influence regulation of gene expression, proliferation, differentiation and apoptosis (Avila et al., 2004). For instance, the accumulation of the metabolite MTA in melanoma cells and in the extracellular environment, resulted from a lack of methythioadenosine phosphorylase (MTAP) expression in melanoma, influences on cell proliferation of surrounding stroma cells and cell invasiveness (Stevens et al., 2009; Limm et al., 2014). In our work, serum creatinine and ADMA was significantly positively correlated with the IgG M protein and negatively correlated with the survival period of MM patients.

In our work, those 12 SCMs (Figure 8), significantly elevated in AG compared with HC, were all decreased in RG group compared with AG. The correlations between those 12 SCMs and clinical features including IgG M protein level and survival period were analyzed, except for 1-Methyladenosine and PC (18:3/16: 0), 10 SCMs were positively correlated with M protein in serum, which was a common clinical indicator for judging the progression or remission of MM. Furthermore, elevated levels of 8 SCMs of 3-Dehydroxycarnitine, DL-Glutamate, Creatinine, N-Acetylputrescine, Butyrylcarnitine, 5′-Methylthioadenosine, Asymmetric dimethylarginine and 1-Methylhistidine have a negative correlation with the survival period of MM, which was firstly reported. Moreover, we firstly reported that metabolite of 5′-Methylthioadenosine might be the independent prognostic factor in MM patients in our work.

In the present study, the identified 23 SCMs were significantly enriched in two metabolic pathways, including arginine and proline metabolism (hsa00330) and glycerophospholipid metabolism (hsa00564). The alteration of arginine and proline metabolic pathways has been identified in human renal cell carcinoma and breast cancer (Geck and Toker, 2016; Lu et al., 2016). Puchades demonstrates elevated serum arginine levels in newly-diagnosed MM patients compared with matched controls (Puchades-Carrasco et al., 2013), while Medriano et al. shows that arginine and proline metabolism is enriched in non-Hodgkin's lymphoma, but not in MM (Medriano et al., 2017). Lysophosphatidiylcholines (LPCs) are up-regulated and phosphatidylcholines (PCs) are down-regulated in ovarian cancer group compared with benign tumor and normal control group and the glycerophospholipid metabolism emerges as a key pathway in ovarian cancer (Zhang Y. et al., 2016).

In the development of MM, the NF-κB and PI3K-AKT-mTOR signaling pathways are activated, which induce bone marrow stromal cells to secrete abundant IL-6 and growth factors, contributing to the growth, proliferation and survival of myeloma cells. NF-κB is the primary signaling pathway in MM, and the targeted drug bortezomib inhibits tumor growth of MM by blocking the NF-κB pathway. Furthermore, p53 deficiency and continuous increase of myeloma growth factors could influence the mTOR signaling pathway by changing the metabolic processes of tumor cells, promoting tumor progression and drug resistance in MM (Falank et al., 2016; Manier et al., 2017). The crosslink among abnormal Glycine, serine and threonine metabolism, Glycerophospholid metabolism, and cell signaling pathways including NF-κB and PI3K-AKT-mTOR signaling pathways might play vital roles in promoting cell proliferation, disturbing cell cycle and inhibiting cell apoptosis of MM.

There are limitations in our study. Firstly, the abundance of 8 SCMs shown in Figure 9 correlated with overall survival of MM patients, but the correlations between SCMs and treatment response were not explored on account of the limitated sample size of responding patients and non-responding patients. Secondly, the difference of metabolite profiling between NDMM and RMM was unable compared due to the small sample size of NDMM and RMM. Last but not at least, 12 SCMs had the diagnostic value in distinguishing plasma samples of AG patients from RG patients and the abundance of 8 out of those 12 SCMs negatively correlated with the overall survival (OS) of patients in Cohort 2 (Figures 8, 9). However, the baseline difference among AG and RG might influence on the overall survival (OS), including LDH, β_2_-MG, amount of IgG. In our future work, the metabolite biomarkers for treatment response will be identified, the metabolite profiling of MM patients in different stages including newly-diagnosed MM, relapsed MM, remission MM will be compared, moreover, the correlations between potential SCMs identified in our study and overall survival will be further validated through a large, clinical cohort.

In summary, we identified the aberrantly changed metabolites in serum of MM patients. A total of 23 SCMs were identified in AG group compared with HC. Twelve out of Twenty-three SCM could distinguish AG patients from RG patients. Those abnormal metabolites were significantly enriched in arginine and glycerophospholipid metabolism. Ten out of Twenty-three SCMs were positively correlated with IgG M protein and 8 SCMs were negatively correlated with survival period of MM patients and only 5′-Methylthioadenosine in SCMs was an independent prognostic factor in MM.

Our study indicated that those abnormalities of metabolites and metabolic pathways might play key roles in pathogenesis in MM progress. Those metabolites might be potential, novel biomarkers for active disease in MM.

## Author contributions

The project was designed by TZ and ZH. The samples were collected by LW, JW, and ZH. The experiments were conducted by TZ. The data were analyzed by HD, TZ, LW, BL, and HS. The manuscript was written by HD, TZ, and ZH.

### Conflict of interest statement

The authors declare that the research was conducted in the absence of any commercial or financial relationships that could be construed as a potential conflict of interest.

## Supplementary material

Figure S1 Representative UPLC-MS total ion chromatograms of serum samples from patients in the AG, RG, and HC groups subjects of Discovery phase under ESI^+^ mode. **(A)**, typical metabolic profiling of serum samples of subjects from the AG, RG, and HC groups by C_18_ column chromatography; **(B)**, typical metabolic profiling of serum samples of subjects from AG, RG, and HC groups by HILIC. UPLC-MS, ultra-perfomance liquid chromatography-tandam tandem mass spectrometry; ESI, electrospray ionization; HILIC, hydrophilic interaction liquid chromatography. The HC, RG, and AG groups in Discovery phase included 14, 10, 12 subjects, respectively.

Figure S2 Histogram showing the abundance of SCMs generated from C_18_ column chromatography in AG, RG, and HC groups of Discovery phase. **(A)**, PC(18:0/18:1(11Z)); **(B)**, PC(16:0/18:1(9Z)); **(C)**, LysoPE(18:0/0:0); **(D)**, LysoPE(18:2/(9Z,12Z)/0:0); **(E)**, PC(18:2(9Z,12Z)/16:0); **(F)**, LysoPE(20:4(8Z,11Z,14Z,17Z)/0:0); **(G)**, PC(20:4(8Z,11Z,14Z,17Z)/18:2(9Z,12Z)); **(H)**, PC(16:0/20:4(8Z,11Z,14Z,17Z)). ^*^indicates *P* < 0.05, ^**^indicates *P* < 0.01, and ^***^indicates *P* < 0.001. *P* < 0.05 indicates statistical significance. SCMs, significantly changed metabolites. The HC, RG, and AG groups in Discovery phase included 14, 10, 12 subjects, respectively.

Figure S3 Histogram showing the abundance of SCMs generated from HILIC column chromatography in AG, RG, and HC groups of Discovery phase. **(A)**, 3-Dehydroxylcarnitine; **(B)**, L-proline; **(C)**, N-Acetylornithine; **(D)**, L-Kynurenine; **(E)**, Ornithine; **(F)**, L-Phenylalanine; **(G)**, Glycine; **(H)**, L-Histidine; **(I)**, L-Glutamine; **(J)**, Pyroglutamic acid. ^*^indicates *P* < 0.05, ^**^indicates *P* < 0.01, and ^***^indicates *P* < 0.001. *P* < 0.05 indicates statistical significance. SCMs, significantly changed metabolites. The HC, RG, and AG groups in Discovery phase included 14, 10, 12 subjects, respectively.

Figure S4 Validation of the abundance of PC(18:0/18:2(9Z,12Z)) generated from C_18_ column chromatography in AG, RG, and HC groups in Validation phase. ^*^indicates *P* < 0.05, ^**^indicates *P* < 0.01, and ^***^indicates *P* < 0.001. *P* < 0.05 indicates statistical significance. SCMs, significantly changed metabolites. Targeted metabolomics analyses was used to validate the abundance of SCMs in Validation phase. The HC, RG, and AG groups in Validation phase included 20, 26, 29 subjects, respectively.

Figure S5 Validation of the abundance of SCMs generated from HILIC column chromatography in AG, RG, and HC groups in Validation phase. **(A)**, DL-Glutamate; **(B)**, L-Octanoylcarnitine; **(C)**, Decanoylcarnitine; **(D)**, 3-Dehydroxycarnitine; **(E)**, L-Proline. ^*^indicates *P* < 0.05, ^**^indicates *P* < 0.01, and ^***^indicates *P* < 0.001. *P* < 0.05 indicates statistical significance. SCMs, significantly changed metabolites. Targeted metabolomics analyses were used to validate the abundance of SCMs in Validation phase. The HC, RG, and AG groups in Validation phase included 20, 26, 29 subjects, respectively.

Table S1 The gradient conditions for C_18_ separation of lipid.

Table S2 The gradient conditions for HILIC separation of polar molecules.

Table S3 The SCMs in AG compared with HC group based on C18 column.

Table S4 The SCMs in AG compared with HC based on HILIC column.

Table S5 The enriched pathway.
