# Supplementary figures and images for: Analysis of the Metabolic Characteristics of Serum Samples in Patients With Multiple Myeloma
Source: Front Pharmacol. 2018 Aug 22;9:884. doi: 10.3389/fphar.2018.00884 (PMC6113671; doi:10.3389/fphar.2018.00884)

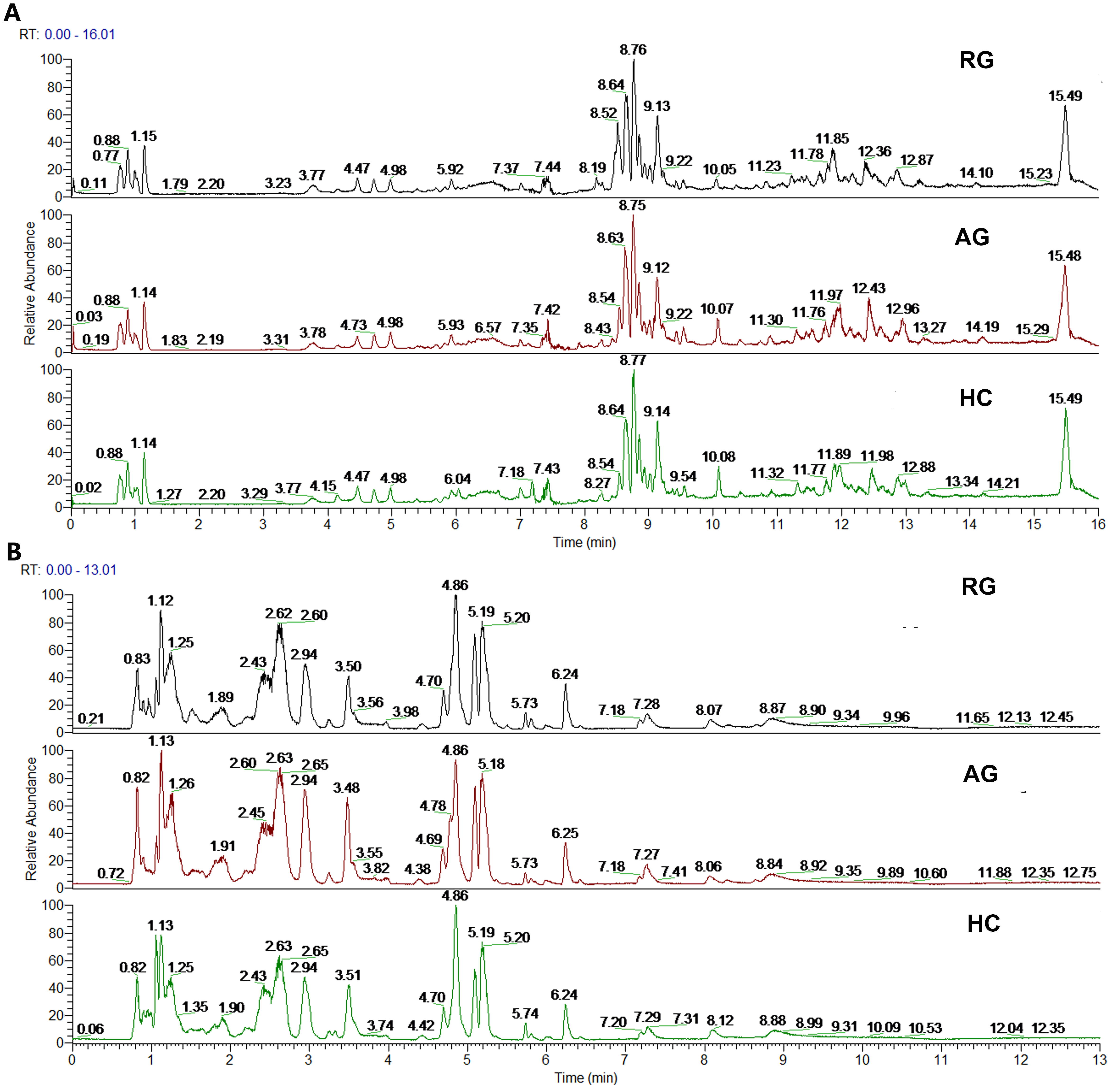

Supplement: Figure S1 — Representative UPLC-MS total ion chromatograms of serum samples from patients in the AG, RG, and HC groups subjects of Discovery phase under ESI+ mode. (A), typical metabolic profiling of serum samples of subjects from the AG, RG, and HC groups by C18 column chromatography; (B), typical metabolic profiling of serum samples of subjects from AG, RG, and HC groups by HILIC. UPLC-MS, ultra-perfomance liquid chromatography-tandam tandem mass spectrometry; ESI, electrospray ionization; HILIC, hydrophilic interaction liquid chromatography. The HC, RG, and AG groups in Discovery phase included 14, 10, 12 subjects, respectively. [file Image_1.tif]

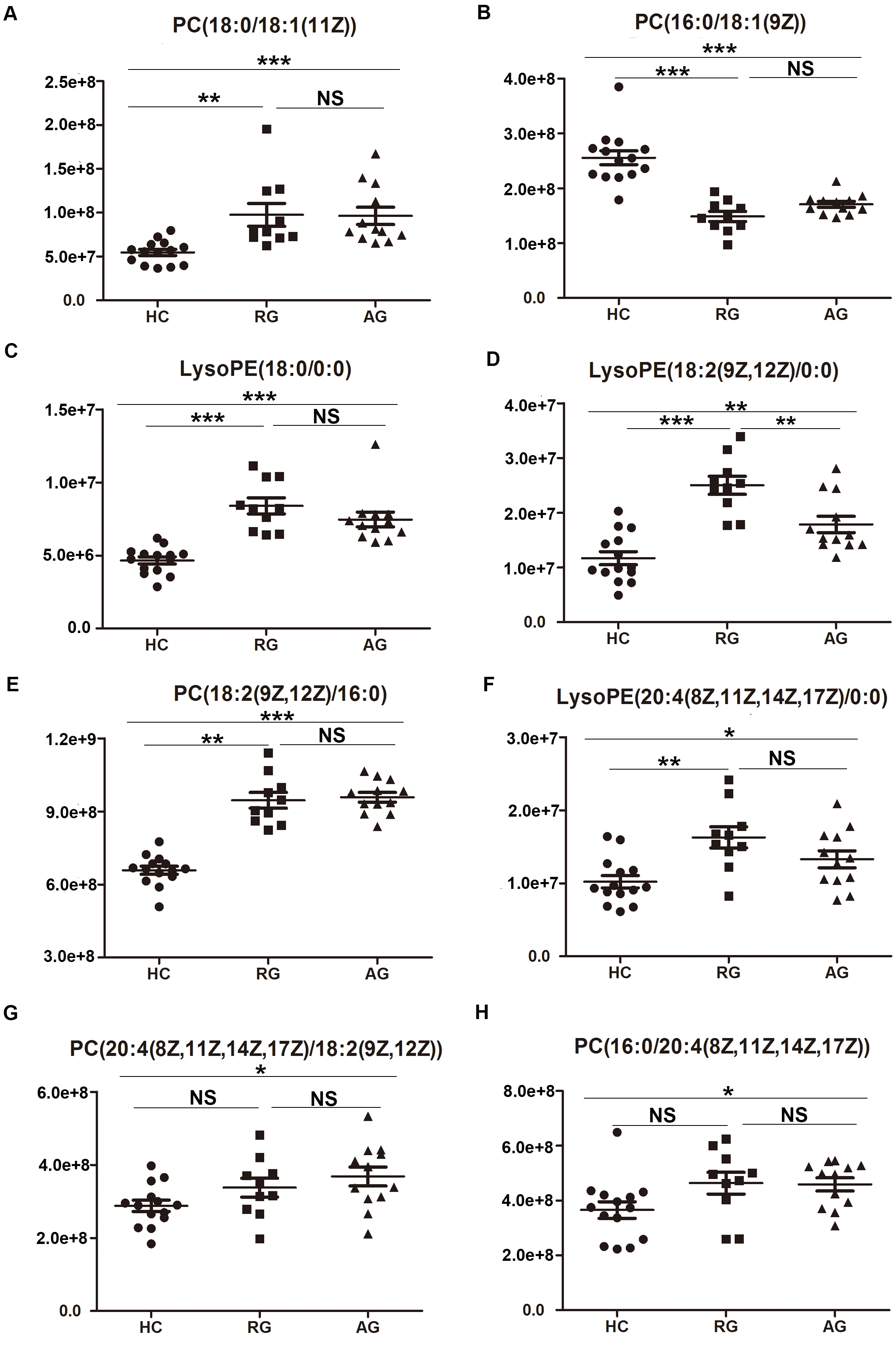

Supplement: Figure S2 — Histogram showing the abundance of SCMs generated from C18 column chromatography in AG, RG, and HC groups of Discovery phase. (A), PC(18:0/18:1(11Z)); (B), PC(16:0/18:1(9Z)); (C), LysoPE(18:0/0:0); (D), LysoPE(18:2/(9Z,12Z)/0:0); (E), PC(18:2(9Z,12Z)/16:0); (F), LysoPE(20:4(8Z,11Z,14Z,17Z)/0:0); (G), PC(20:4(8Z,11Z,14Z,17Z)/18:2(9Z,12Z)); (H), PC(16:0/20:4(8Z,11Z,14Z,17Z)). *indicates P < 0.05, **indicates P < 0.01, and ***indicates P < 0.001. P < 0.05 indicates statistical significance. SCMs, significantly changed metabolites. The HC, RG, and AG groups in Discovery phase included 14, 10, 12 subjects, respectively. [file Image_2.tif]

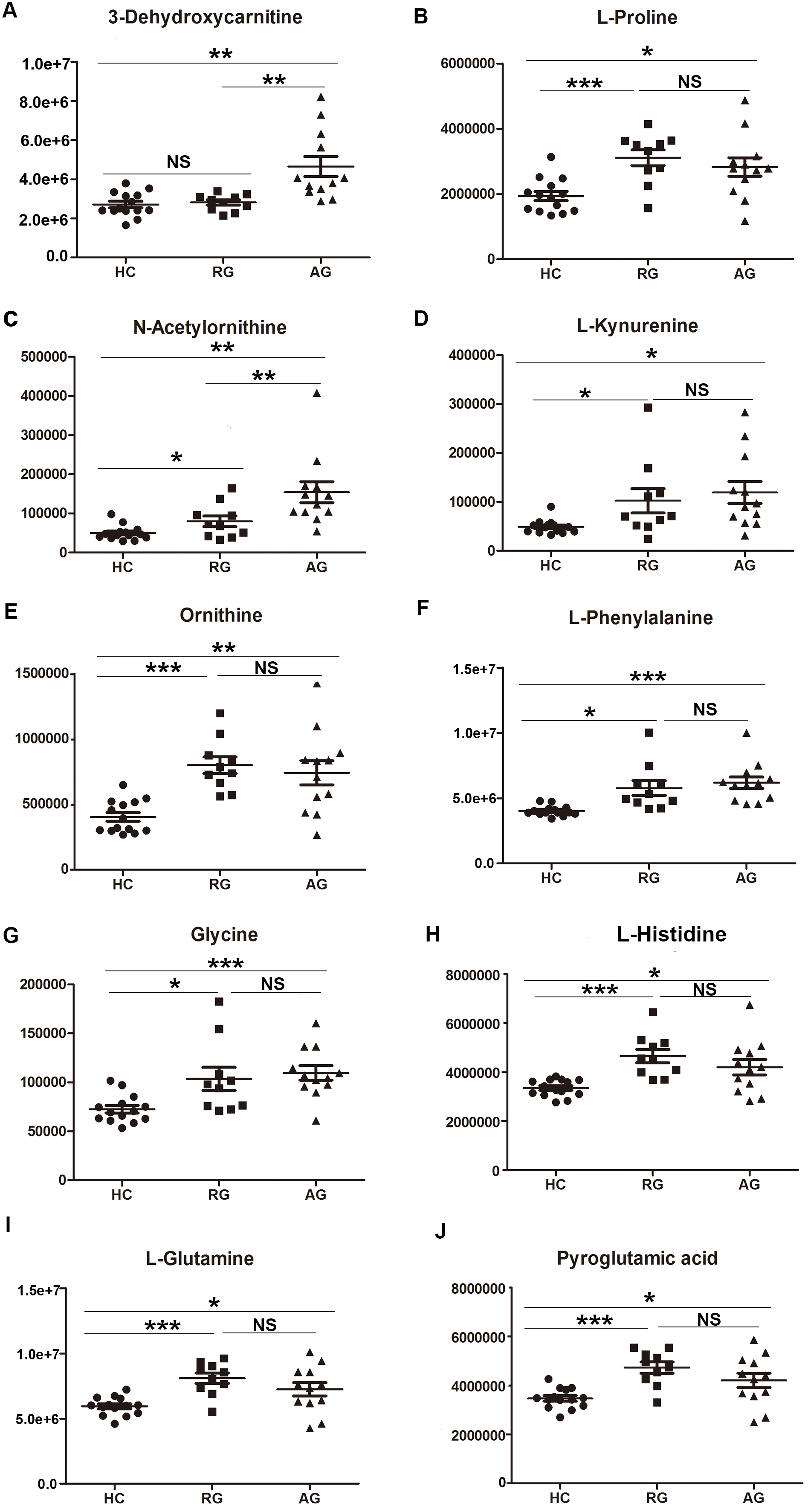

Supplement: Figure S3 — Histogram showing the abundance of SCMs generated from HILIC column chromatography in AG, RG, and HC groups of Discovery phase. (A), 3-Dehydroxylcarnitine; (B), L-proline; (C), N-Acetylornithine; (D), L-Kynurenine; (E), Ornithine; (F), L-Phenylalanine; (G), Glycine; (H), L-Histidine; (I), L-Glutamine; (J), Pyroglutamic acid. *indicates P < 0.05, **indicates P < 0.01, and ***indicates P < 0.001. P < 0.05 indicates statistical significance. SCMs, significantly changed metabolites. The HC, RG, and AG groups in Discovery phase included 14, 10, 12 subjects, respectively. [file Image_3.tif]

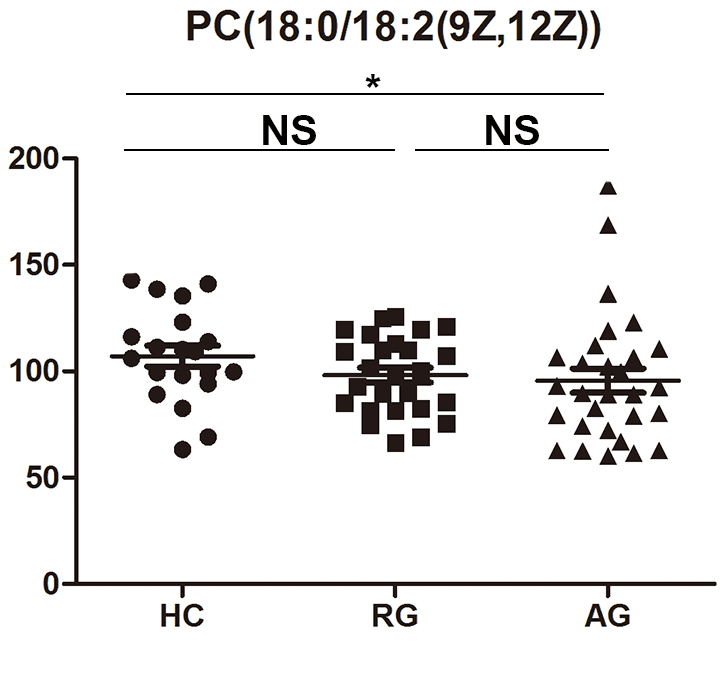

Supplement: Figure S4 — Validation of the abundance of PC(18:0/18:2(9Z,12Z)) generated from C18 column chromatography in AG, RG, and HC groups in Validation phase. *indicates P < 0.05, **indicates P < 0.01, and ***indicates P < 0.001. P < 0.05 indicates statistical significance. SCMs, significantly changed metabolites. Targeted metabolomics analyses was used to validate the abundance of SCMs in Validation phase. The HC, RG, and AG groups in Validation phase included 20, 26, 29 subjects, respectively. [file Image_4.tif]

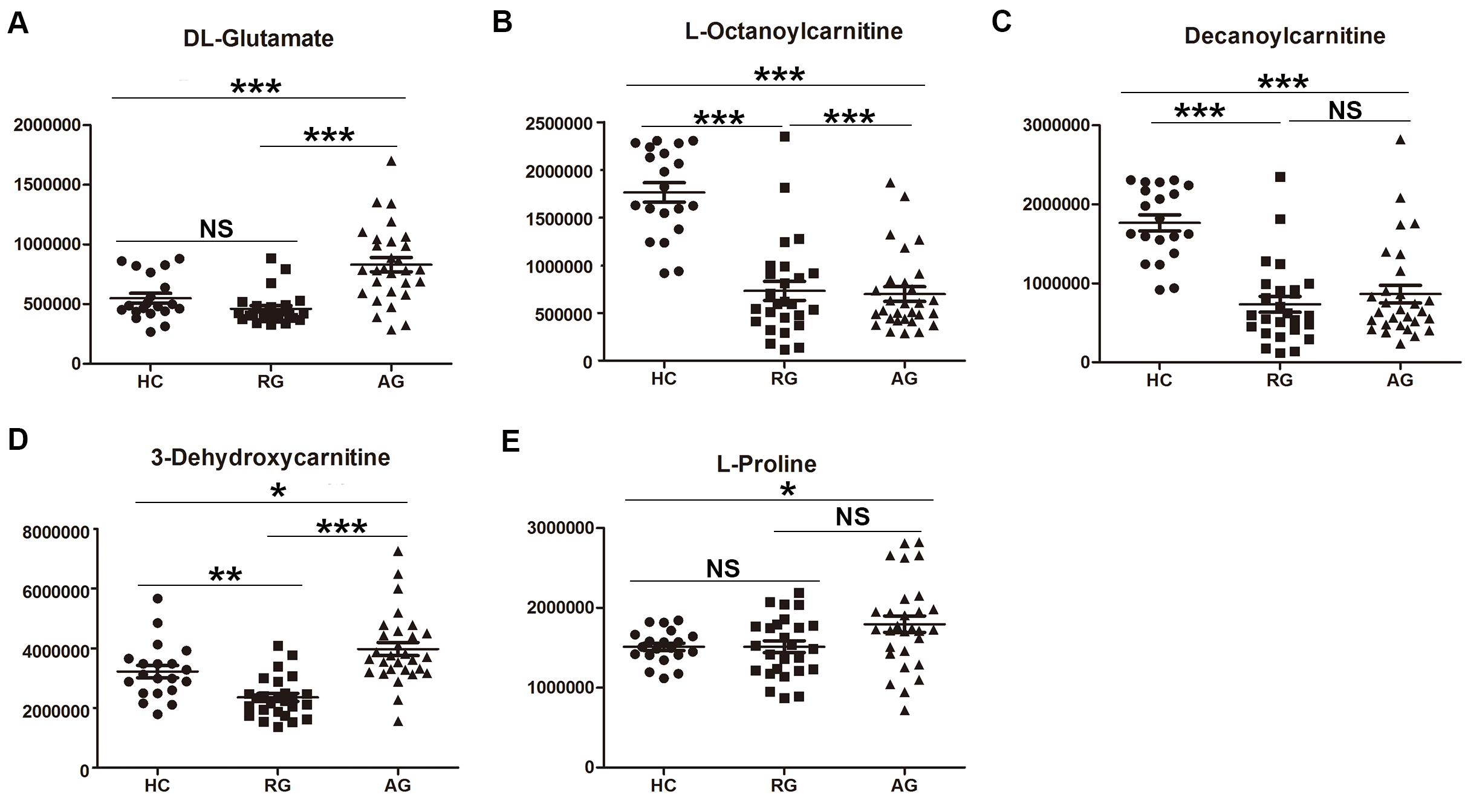

Supplement: Figure S5 — Validation of the abundance of SCMs generated from HILIC column chromatography in AG, RG, and HC groups in Validation phase. (A), DL-Glutamate; (B), L-Octanoylcarnitine; (C), Decanoylcarnitine; (D), 3-Dehydroxycarnitine; (E), L-Proline. *indicates P < 0.05, **indicates P < 0.01, and ***indicates P < 0.001. P < 0.05 indicates statistical significance. SCMs, significantly changed metabolites. Targeted metabolomics analyses were used to validate the abundance of SCMs in Validation phase. The HC, RG, and AG groups in Validation phase included 20, 26, 29 subjects, respectively. [file Image_5.tif]
