# Supplementary material for: Analysis of the Metabolic Characteristics of Serum Samples in Patients With Multiple Myeloma
Source: Front Pharmacol. 2018 Aug 22;9:884. doi: 10.3389/fphar.2018.00884 (PMC6113671; doi:10.3389/fphar.2018.00884)
Supplement: Table S2 — The gradient conditions for HILIC separation of polar molecules. [file Table_2.DOCX]

**Table S2 The gradient conditions for HILIC separation of polar molecules**

| **Total time (min)** | **A (v %)** | **B (v %)** |
| --- | --- | --- |
| 0 | 95 | 5 |
| 1 | 95 | 5 |
| 10 | 50 | 50 |
| 12 | 50 | 50 |
| 12.1 | 95 | 5 |
| 16 | 95 | 5 |

min: minute; HILIC: hydrophilic interaction liquid chromatography.
