# Supplementary material for: Analysis of the Metabolic Characteristics of Serum Samples in Patients With Multiple Myeloma
Source: Front Pharmacol. 2018 Aug 22;9:884. doi: 10.3389/fphar.2018.00884 (PMC6113671; doi:10.3389/fphar.2018.00884)
Supplement: Table S3 — The SCMs in AG compared with HC group based on C18 column. [file Table_3.DOCX]

**Table S3 The SCMs in AG compared with HC group based on C18 column**

| **HMDB ID** | **Description** | **Formula** | **RT(min)** | ***P*-value** | **VIP** | **FC** |
| --- | --- | --- | --- | --- | --- | --- |
| HMDB08166 | PC(18:3(6Z,9Z,12Z)/16:0) | C42H78NO8P | 12.451 | 1.52E-07 | 1.297 | 2.783 |
| HMDB00564 | PC(16:0/16:0) | C40H80NO8P | 12.429 | 0.02770579 | 1.153 | 2.630 |
| HMDB11473 | LysoPE(0:0/16:0) | C21H44NO7P | 8.689 | 0.00023959 | 1.221 | 2.617 |
| HMDB08034 | PC(18:0/16:0) | C42H84NO8P | 13.538 | 4.73E-06 | 1.275 | 2.084 |
| HMDB08037 | PC(18:0/18:1(11Z)) | C44H86NO8P | 13.775 | 5.67E-05 | 1.249 | 1.762 |
| HMDB11503 | LysoPE(16:0/0:0) | C21H44NO7P | 8.778 | 0.0003468 | 1.207 | 1.744 |
| HMDB11505 | LysoPE(18:1(11Z)/0:0) | C23H46NO7P | 8.859 | 0.00289927 | 1.157 | 1.714 |
| HMDB07972 | PC(16:0/18:1(9Z)) | C42H82NO8P | 12.629 | 4.12E-08 | 1.301 | 1.672 |
| HMDB11130 | LysoPE(18:0/0:0) | C23H48NO7P | 9.144 | 5.12E-06 | 1.268 | 1.601 |
| HMDB13464 | SM(d18:0/16:1(9Z)) | C39H79N2O6P | 11.466 | 8.08E-09 | 1.456 | 1.580 |
| HMDB11507 | LysoPE(18:2(9Z,12Z)/0:0) | C23H44NO7P | 8.640 | 0.00239775 | 1.169 | 1.528 |
| HMDB08039 | PC(18:0/18:2(9Z,12Z)) | C44H84NO8P | 12.977 | 1.60E-06 | 1.283 | 1.482 |
| HMDB10390 | LysoPC(20:0) | C28H58NO7P | 9.576 | 0.03985958 | 1.142 | 1.458 |
| HMDB08133 | PC(18:2(9Z,12Z)/16:0) | C42H80NO8P | 11.970 | 9.23E-11 | 1.596 | 1.456 |
| HMDB10383 | LysoPC(16:1(9Z)) | C24H48NO7P | 8.520 | 0.00106326 | 1.182 | 1.396 |
| HMDB11518 | LysoPE(20:4(8Z,11Z,14Z,17Z)/0:0) | C25H44NO7P | 8.627 | 0.03764844 | 1.146 | 1.302 |
| HMDB11128 | LysoPC(0:0/18:0) | C26H54NO7P | 9.015 | 0.00489521 | 1.152 | 1.295 |
| HMDB08467 | PC(20:4(8Z,11Z,14Z,17Z)/18:2(9Z,12Z)) | C46H80NO8P | 11.765 | 0.01531087 | 1.179 | 1.279 |
| HMDB07983 | PC(16:0/20:4(8Z,11Z,14Z,17Z)) | C44H80NO8P | 11.889 | 0.02055987 | 1.159 | 1.256 |
| HMDB13122 | LysoPC(P-18:0) | C26H54NO6P | 9.318 | 0.04844716 | 1.138 | 1.245 |

SCM: significantly changed metabolite; AG: aggressive group; HC: healthy controls; RT: Retention time; FC: fold change; VIP: variable importance in the projection
