# Supplementary material for: Analysis of the Metabolic Characteristics of Serum Samples in Patients With Multiple Myeloma
Source: Front Pharmacol. 2018 Aug 22;9:884. doi: 10.3389/fphar.2018.00884 (PMC6113671; doi:10.3389/fphar.2018.00884)
Supplement: Table S4 — The SCMs in AG compared with HC based on HILIC column. [file Table_4.DOCX]

**Table S4 The SCMs in AG compared with HC based on HILIC column**

| **ID** | **Description** | **Formula** | **RT(min)** | **P-value** | **VIP** | **FC** |
| --- | --- | --- | --- | --- | --- | --- |
| HMDB13161 | 2-Hexenoylcarnitine | C13H23NO4 | 2.182 | 0.001 | 1.15706 | 7.898 |
| HMDB00001 | 1-Methylhistidine | C7H11N3O2 | 7.075 | 0.000 | 1.61659 | 7.578 |
| HMDB01539 | Asymmetric dimethylarginine | C8H18N4O2 | 6.652 | 0.000 | 1.33611 | 6.632 |
| HMDB01173 | 5'-Methylthioadenosine | C11H15N5O3S | 1.504 | 0.000 | 1.65817 | 4.739 |
| HMDB02013 | Butyrylcarnitine | C11H21NO4 | 2.538 | 0.000 | 1.18087 | 4.228 |
| HMDB02064 | N-Acetylputrescine | C6H14N2O | 4.767 | 0.000 | 1.6289 | 3.932 |
| HMDB13128 | Valerylcarnitine | C12H23NO4 | 2.253 | 0.001 | 1.07011 | 3.638 |
| HMDB03357 | N-Acetylornithine | C7H14N2O3 | 6.006 | 0.000 | 1.58006 | 3.101 |
| HMDB03331 | 1-Methyladenosine | C11H15N5O4 | 5.310 | 0.000 | 1.67385 | 2.510 |
| HMDB00684 | L-Kynurenine | C10H12N2O3 | 4.816 | 0.001 | 1.26113 | 2.433 |
| HMDB60475 | DL-Glutamate | C5H9NO4 | 6.095 | 0.000 | 1.3583 | 2.183 |
| HMDB00562 | Creatinine | C4H7N3O | 2.957 | 0.000 | 1.32346 | 2.112 |
| HMDB00214 | Ornithine | C5H12N2O2 | 7.356 | 0.001 | 1.23664 | 1.837 |
| HMDB00651 | Decanoylcarnitine | C17H33NO4 | 1.624 | 0.013 | 1.02428 | 1.804 |
| HMDB06831 | 3-Dehydroxycarnitine | C7H15NO2 | 3.590 | 0.000 | 1.26784 | 1.718 |
| HMDB00791 | L-Octanoylcarnitine | C15H29NO4 | 1.776 | 0.029 | 1.15686 | 1.621 |
| HMDB00159 | L-Phenylalanine | C9H11NO2 | 4.718 | 0.000 | 1.45334 | 1.536 |
| HMDB00123 | Glycine | C2H5NO2 | 6.046 | 0.000 | 1.28024 | 1.510 |
| HMDB00162 | L-Proline | C5H9NO2 | 5.275 | 0.009 | 1.2169 | 1.457 |
| HMDB00177 | L-Histidine | C6H9N3O2 | 7.289 | 0.011 | 1.20606 | 1.255 |
| HMDB00641 | L-Glutamine | C5H10N2O3 | 6.242 | 0.037 | 1.20329 | 1.219 |
| HMDB00267 | Pyroglutamic acid | C5H7NO3 | 6.242 | 0.039 | 1.20309 | 1.213 |

RT: retention time; FC: fold change; VIP: variable importance in the projection
