# Supplementary material for: Analysis of the Metabolic Characteristics of Serum Samples in Patients With Multiple Myeloma
Source: Front Pharmacol. 2018 Aug 22;9:884. doi: 10.3389/fphar.2018.00884 (PMC6113671; doi:10.3389/fphar.2018.00884)
Supplement: Table S5 — The enriched pathway. [file Table_5.DOCX]

**Table S5 The enriched pathway**

| **Metabolic pathway** | **Total** | **Hits** | ***P*-value** | **SCMs** |
| --- | --- | --- | --- | --- |
| Arginine and proline metabolism | 77 | 3 | 0.0056 | L-Proline,Creatinine,N-Acetylputrescine |
| Glycerophospholipid metabolism | 39 | 2 | 0.0152 | LysoPC(18:1(9Z)),Phosphatidylcholine |

SCM: significantly changes metabolites.
